# An economic evaluation of the MultICath randomised controlled trial, comparing combined use of reusable and single-use intermittent catheters to single-use catheters only

**DOI:** 10.1186/s12962-026-00750-z

**Published:** 2026-05-09

**Authors:** Sara McCloskey, Tracey H. Sach, Margaret Macaulay, Miriam R. Avery, Thomas J. Chadwick, Bridget Clancy, Sylvia Dickson, Nicola Goudie, Karen Guerrero, Suzanne Hagen, Cathy Murphy, Gillian C. Watson, Nina Wilson, Ruth Wood, Mandy Fader

**Affiliations:** 1 School of Primary Care, Population Sciences and Medical Education, University of Southampton, University Road, Southampton, SO17 1BJ UK; 2 School of Health Sciences, University of Southampton, University Road, Southampton, SO17 1BJ UK; 3 Newcastle Clinical Trials Unit, Newcastle University, Newcastle Upon Tyne, NE1 7RU UK; 4 Nurses, Midwives and Allied Health Professionals Research Unit, Glasgow Caledonian University, Cowcaddens Road, Glasgow, G4 0BA Scotland, UK; 5 Department of Urogynaecology, Maternity Building, Queen Elizabeth University Hospital, 1345 Govan Road, Glasgow, G51 4TF Scotland, UK; 6 School of Primary Care, Population Sciences and Medical Education, University of Southampton, Aldermoor Health Centre, Southampton, S016 5ST UK

**Keywords:** Single-use catheters, Multi-use catheters, Intermittent catheterization, Economic evaluation, Cost-utility, D61, I10

## Abstract

**Objectives:**

Widespread adoption of single-use intermittent catheters for bladder drainage has led to increased costs and environmental waste. Reusable catheters could reduce both. The MultICath study showed that combined reusable and single-use catheters (Mixed-use) was non-inferior to single-use catheters for urinary tract infection and quality of life, but the economic impact is unknown. We aim to determine whether Mixed-use is cost-effective compared to Single-use only.

**Methods:**

Cost-utility and cost-consequence analyses were conducted alongside MultICath. The analysis took the United Kingdom payer perspective, using individual patient-level data of the 578 randomised (1:1) trial participants over 12 months. Costs included catheter and cleaning costs, antibiotic costs, and health-related visit costs. Outcomes were measured in quality adjusted life years (QALYs), estimated from EQ-5D-5L data. Incremental, sensitivity, and scenario analyses were conducted.

**Results:**

Mixed-use participants used on average 902 (95% CI 755.13; 1049.31) fewer single-use catheters per annum than Single-use participants. Mixed-use was cost-effective in all analyses. Base-case annual incremental cost savings were -£1348.82 (95% CI -1939.98; -757.65) whilst incremental QALYs were negligible (-0.001; [-0.026; 0.024]). These estimates produced a positive net monetary benefit of £1328.82 at a willingness-to-pay threshold of £20,000. The probability of Mixed-use being cost-effective was never below 96.6%. The primary limitation was differential withdrawal rates between groups, accounted for in sensitivity analyses.

**Conclusions:**

Mixed-use is cost-effective for the UK National Health Service, and provision of reusable catheters should be considered. These findings are also relevant for other health services worldwide with high single-use catheter costs.

**Supplementary Information:**

The online version contains supplementary material available at 10.1186/s12962-026-00750-z.

## Introduction

Intermittent catheterisation (IC) is the treatment of choice for long-term management of incomplete bladder drainage [1], and the number of people carrying out IC is increasing [2]. IC users typically catheterise four to six times per day. There has been widespread adoption of single-use catheters, as opposed to traditional reusable catheters [3], worldwide over the last three decades, driven primarily by concerns about urinary tract infection and patient quality of life [4]. 

In the UK, it is estimated that 75 million IC catheters are used annually, costing the NHS approximately £165 million in 2022 [5]. These figures reflect single-use catheter utilisation and costs as in the UK all catheters are packed and labelled as single use [6]. In the United States of America (USA), Medicare alone spent $407 million on IC catheters in 2020 [7], and single-use catheters produce up to 85 million pounds of waste annually [8]. These economic and environmental consequences are particularly concerning given the lack of data to support using single-use catheters instead of reusable catheters [9]. The MultICath clinical trial aimed to address this gap by determining whether a Mixed-use strategy (reusable and single-use catheters) was non-inferior to single-use catheters only with regards to UTI incidence [10].

We conducted an economic evaluation alongside this trial to determine whether Mixed-use may be more cost-effective than Single-use only for the UK NHS. To date, there is limited evidence on the cost-effectiveness of a Mixed-use catheter strategy. However, a 2013 systematic review and decision analytic model found evidence that reusable catheters may be more cost-effective than single-use options in the UK, albeit based on low-quality clinical evidence [11]. The per-protocol analysis of the MultICath randomised controlled trial (RCT) found that Mixed-use (*N* = 171) was non-inferior to Single-use (*N* = 244) with regards to UTI incidence and catheter-related quality of life [10]. Using the individual-participant level data from the MultICath trial, we aim to provide estimates of costs and outcomes to enable evidence-based prescribing of IC catheters.

## Materials and methods

The economic evaluation was conducted according to the Health Economic Analysis Plan (Supplementary Table 6) alongside MultICath, an open-label, pragmatic, parallel-group, non-inferiority randomised controlled trial (RCT). Recruitment occurred between February 2021 and June 2023. Participants were long-term urethral intermittent catheter users aged 18 and above who were approached by their GP practices, nurse prescribing teams or NHS clinics, or people who directly approached the trial team. Written consent was obtained from all participants [10, 12]. Patients and the public were integral to the design of the intervention and development of documentation [12, 13]. 

### Interventions

Participants were randomised (1:1) to the intervention and control group using a secure 24-hour web-based system and stratified by sex and centre (Southampton or Glasgow). The control group used exclusively single-use catheters, while the intervention group adopted a Mixed-use strategy, consisting of single-use and reusable catheters. The Mixed-use group was instructed to use their reusable catheter as often as possible, and at least daily, and to use the single-use catheters as needed. Mixed-use was chosen instead of reuse only because patient input indicated that using reusable catheters might not be feasible for every catheterisation, such as when catheterising outside their home. Further, Cliny catheters are made of silicone, which provides a different catheterisation experience that might not suit all users, making the need for patient choice key [6].

Single-use catheters were prescribed as usual through the NHS. Mixed-use participants were supplied with Cliny^®^ reusable all-silicone catheters (Create Medic Ltd), lubricant, cleaning solution (Milton), a cleaning container, and balloon syringe and given a two-week learning period to adapt to the reusable catheter. After this learning period, both groups had a 12-month follow-up period. Previous work has shown that the Cliny catheters and cleaning method are acceptable to users [10, 13].

### Resource use and costs

Participants reported the number of single-use catheterisations they performed in a seven-day period in monthly diaries (Supplementary Table 6). It was assumed that catheter use in each seven-day period was reflective of monthly use, and use for this period was extrapolated out to the month. If participants recorded their single-use catheter use for between one and six days (0.2% of observations), their monthly average was calculated from the completed days. For single-use catheters, the monthly number of catheterisations per participant was multiplied by £1.69, the average price of a Nelaton catheter on the 2023/24 Prescription Cost Analysis (PCA) [14]. 

Mixed-use costs were calculated by multiplying the cost of any intervention supplies by the number of items that a participant required, as recorded in the monthly diary. These additional costs were added to the £48 worth of reusable catheter supplies (4 catheters, 1 kit), deflated to their 2023/24 equivalent using the Consumer Price Index (CPI) [15], £13.49 of cleaning supplies (1 container, 1 syringe, and 24 Milton tablets), and one tube of OptiLube that participants were given at the start of the study. The intervention was designed to be self-taught, so implementation costs were not needed.

UTI-related antibiotic use was recorded in the monthly diary if a participant had a UTI that month. Antibiotics were costed at their respective prices as found in the 2023/24 PCA [14].

Healthcare resource use data (Supplementary Table 6) was collected separate from the monthly diaries at baseline, six-months, and 12-month. These questionnaires asked about all primary, secondary, and emergency care that the patients underwent during that period. Participants were also asked about care they paid for out-of-pocket, which was incorporated into a sensitivity analysis. There was no data on how participants paid for their NHS catheter prescriptions, such as paying the prescription charge out-of-pocket, having a pre-paid certificate, or having a medical exemption. Therefore, out-of-pocket costs associated with the prescription of catheters on the NHS were not included. Visits were costed according to the 2023/24 NHS [16] and Personal Social Services (PSS) [17] reference costs (Table 1).

**Table 1:** Reference costs in 2023/24 UK pounds sterling

Cost Item	Unit Cost (£)	Source
**Intervention**		
Milton© (per tablet)	£0.02	2025 commercial list price deflated [15] to 2023/24 £s
Milton© cleaning container	£11	2023/24 commercial list price [13]
Balloon syringe for cleaning	£2.49	2023/24 commercial list price [13]
Cliny^®^ intermittent catheterisation kits (catheter + case + carrying pouch)	£19.65	2025 commercial list price deflated [15] to 2023/24 £s
Additional Cliny^®^ catheters (per pack of 5)	£34.40	2025 commercial list price deflated [15] to 2023/24 £s
Optilube lubricating jelly (82 g tube)	£1.79	2023/24 Drug Tariff [18]
Single-use catheters	£1.69	2023/24 PCA [14]; average price of all Nelaton single use catheters
**Health Care Resource Use**		
GP visits	£37	2023/2024 PSSRU reference costs [17]
Practice Nurse visit	£7.83	2023/24 PSSRU reference cost [17]; assuming 10 min visit
Hospital Doctor at practice	£163	2023/24 NHS reference cost [16]; assuming Urologist follow up visit
Hospital Doctor phone consultation	£124	2023/24 NHS reference costs [16]; assuming Urologist follow up visit
A&E	£273	2023/24 NHS reference costs [16]; weighted average of all emergency care
Inpatient	£2,427	2023/24 NHS reference costs [16]; weighted average of all inpatient cases for kidney or urinary tract infection
**Medication**		
Average cost per day of all antibiotics used in trial	£4.45	2023/24 PCA [14]

### Outcomes

Quality adjusted life years (QALYs; a measure of health benefit that combines both the length of life and its quality) were the primary outcome for the health economic evaluation. Health-related quality of life (HRQoL) data were gathered from participants using the EQ-5D-5L [19] at baseline and every three months thereafter and mapped to utility values using the approach recommended by NICE at the time of analysis [20]. Linear interpolation and area under the curve analysis were used to estimate QALYs from the EQ-5D-5L questionnaire [21]. 

### Primary economic analysis

The primary analysis was a cost-utility analysis to determine the cost-effectiveness of Mixed-use compared to the Single-use over a twelve-month time horizon from the perspective of the NHS and PSS. The analysis focused on estimating the incremental costs and incremental QALYs associated with the intervention, as well as the percentage of time the intervention was cost-effective at willingness-to-pay thresholds of £20,000 and £30,000. Costs and outcomes were not discounted given the twelve-month time horizon. Additionally, costs and outcomes were presented descriptively as a cost-consequence analysis.

To be consistent with the clinical analysis, the primary analysis was conducted per-protocol (PP). PP was defined as “all randomised participants who completed at least 6 months data collection and, for those in the Mixed-use group, used reusable catheters for at least 6 months, had a percentage reuse of at least 25% or an average of 0.75 times per day (Mixed-use minimum threshold), and who completed 80% of their catheter diaries.” [10].

Seemingly unrelated regressions with non-parametric bootstrapping (10,000 simulations) were used to determine the incremental costs and incremental QALYs for the cost-utility analysis. These methods were chosen because costs and QALYs were both skewed and correlated with each other [22]. Unadjusted and adjusted analyses were conducted, with the adjusted analysis controlling for baseline cost, baseline utility, sex, age, Barthel index, antibiotic prophylaxis, time in study, centre, antibiotic use at baseline, and catheter use at baseline. Results are presented as mean net monetary benefit (NMB), as well as displayed on cost-effectiveness acceptability curves and on the incremental cost-effectiveness plane (Supplementary Figs. 1 & 2).

Missing data were assumed to be missing at random and multiple imputation was used. The imputation model contained the same covariates as the adjusted model, as well as antibiotic cost at three time periods that were found to significantly predict missing cost and utility data [23]. Two imputation models were run, the first of which was for utility at each time, total visit costs, and total antibiotic costs. The second model was for total catheter costs. Catheter costs were imputed separately because people who withdrew for the Mixed-use group would revert to Single-use only, so the sample for imputing catheter costs was restricted to the control group.

### Secondary economic analyses

In addition to the base-case PP analysis, the same analysis was conducted for the modified intention-to-treat (mITT) and intention-to-treat (ITT) populations. The mITT population included all randomised participants who remained in the trial for at least six months, whereas the ITT population includes all randomised participants.

Subgroup analyses were conducted for sex and baseline Barthel index (</=89 or > 89) in the mITT population, to be consistent with the clinical analysis. A threshold analysis was done to assess the impact of changes in the price of single-use catheters on the findings. Other sensitivity analyses, conducted on the PP population, included a complete case analysis to explore the impact of missing data and broadening the perspective by including participants’ out-of-pocket expenses.

## Results

### Study population

The PP population consisted of 244 participants in the Single-use group and 171 participants in the Mixed-use group. The mITT sample had 244 participants in the Single-use group and 207 in the Mixed-use group. The full trial had 578 participants, with 289 in each group. 29.7% of the Mixed-use group withdrew from the trial, whereas the Single-use group had a withdrawal rate of 15.6%. Demographic characteristics were balanced across groups in all populations.

### Resource use and costs

Mean resource use is shown in Table 2 and unadjusted mean costs are shown in Table 3. Most notably, Mixed-use was £1682.85 cheaper per participant than Single-use, driven primarily by the difference in catheter costs. While the mean intervention costs were only accrued by the Mixed-use group, this was offset by the significant reduction in single-use catheter costs. Additionally, the Mixed-use group had significantly fewer people who used an antibiotic during the trial, as well as significantly lower GP visit costs. There were no significant differences in costs across any other domains. These findings were consistent with the ITT population (Supplementary Tables 1 & 2).

### Outcomes

Mean unadjusted utility values and QALYs for the available cases within the PP population are shown in Table 4. All participants had EQ-5D-5L data available at baseline. 160/171 (93.6%) participants in the intervention group and 230/244 (94.3%) participants in the control group had complete EQ-5D-5L data across the trial. The Mixed-use group had higher utility values at all time points, including at baseline. These findings were consistent with the EQ-5D VAS, as well as in the ITT analysis (Supplementary Table 3). Baseline differences were adjusted for in the incremental analyses.

**Table 2:** Mean (Standard Deviation) resource use and mean difference (95% Confidence Interval) per patient over 12 months for the mixed-use strategy compared to single-use only strategy

	Mixed-use strategy (*n* = 171)		Single-use catheters only strategy (*n* = 244)		Mean difference
	Mean (*n*)	Std dev	Mean (*n*)	Std dev	(95% CI)
**Intervention (number)**					
Cliny^®^ catheters	9.70 (*n* = 161)	5.35	0.00 (*n* = 224)	0.00	9.70 (9.02; 10.37)
Cliny^®^ kits	3.83 (*n* = 161)	4.22	0.00 (*n* = 224)	0.00	3.83 (3.29; 4.36)
Single-use catheters	625.40 (*n* = 155)	605.56	1527.62 (*n* = 224)	783.23	-902.22 (-1049.31; -755.13)
**Primary**,** secondary**,** and emergency care (number)**					
GP visits	2.09 (*n* = 162)	2.93	2.95 (*n* = 235)	4.27	-0.86 (-1.62; -0.10)
Nurse visits	2.25 (*n* = 162)	2.84	3.04 (*n* = 235)	7.82	-0.80 (-2.06; 0.46)
Outpatient visits	3.06 (*n* = 162)	6.06	3.20 (*n* = 235)	4.99	− 0.15 (-1.24; 0.95)
Inpatient stay	0.23 (*n* = 165)	0.61	0.27 (*n* = 237)	0.77	-0.04 (-0.19; 0.20)
A&E visit	0.17 (*n* = 162)	0.44	0.26 (*n* = 235)	0.79	-0.09 (-0.23; 0.04)
Other visits	0.01 (*n* = 162)	0.16	0.04 (*n* = 235)	0.59	-0.03 (-0.11; 0.07)
**Medication (number of people who took at least one antibiotic)**					
Medication – all antibiotics	53 (*n* = 148)	71.20	106 (*n* = 215)	107.74	-53 (-72.87; -33.13)
**Participant personal resource use (number of people)**					
Participant resource use e.g. private appointments, over the counter purchases	74 (*n* = 162)	81.40	107 (*n* = 235)	117.28	-33 (-53.86; -12.14)

**Table 3:** Mean (Standard Deviation) cost and cost difference (95% Confidence Interval) per patient over 12 months for the mixed-use strategy compared to the Single-use only strategy (in 2023/24 UK pounds sterling)

	Mixed-use strategy (*n* = 171)		Single-use catheters only strategy (*n* = 244)		Mean difference
	Mean (*n*)	Std dev	Mean (*n*)	Std dev	(95% CI) £’s
**Intervention**					
Cliny^®^ catheters and accessories (kits, single catheters, Optilube, Milton©, Milton© container, syringe)	£172.80 (*n* = 161)	83.71	0.00	0.00	172.80 (162.27; 183.32)
Single-use catheters	£1056.92 (*n* = 155)	1023.39	£2581.67 (*n* = 224)	1323.66	-£1524.75 (-1773.33; -1276.16)
**Primary**,** secondary**,** and emergency care**					
GP visits	£77.43 (*n* = 162)	108.47	£109.11 (*n* = 235)	158.15	-£31.68 (-59.80; − 3.57;)
Nurse visits	£17.59 (*n* = 162)	22.27	£23.86 (*n* = 235)	61·26	-£6.26 (-16.15; 3.62)
Outpatient visit	£488.91 (*n* = 162)	970.31	£507.69 (*n* = 235)	798.99	-£18.78 (-156.46; 194.03)
Inpatient stay	£558.95 (*n* = 165)	1483.19	£665.63 (*n* = 237)	1877.11	-£106.69 (-450.82; 237.45)
A&E visit	£47.19 (*n* = 162)	120.10	£72.03 (*n* = 235)	216.85	-£24.84 (-61.73; 12.04)
Other visits	£36.67 (*n* = 163)	105.50	£52.68 (*n* = 235)	287.31	-£16.01 (-62.29; 30.27)
**Medication**					
Medication – All antibiotics for UTI treatment	£5.27 (*n* = 148)	28.42	£8.15 (*n* = 215)	19.83	-£2.88 (-7.86; 2.10)
**Participant personal costs**					
Participant resource use e.g. private appointments, over the counter purchases	£224.11 (*n* = 163)	832.36	£691.21 (*n* = 235)	3450.87	-£467.10 (-1009.30; 75.10)
**Total health care costs**,** excluding personal resource use**	**£2271.20 (** ***n*** ** = 145)**	**2044.33**	**£3954.05** **(** ***n*** **= 204)**	**2629.39**	**-£1682.85** **(-2196.43; -1169.28)**

**Table 4:** Per protocol (PP) unadjusted outcomes

	Mixed-use strategy (*n* = 171)		Single-use catheters only strategy (*n* = 244)		Mean difference
	Mean	Std dev	Mean	Std dev	(95% CI)
**EQ-5D-5L Index**					
EQ-5D-5L Baseline	0.825 (*n* = 171)	0.183	0.769 (*n* = 244)	0.221	-0.055 (-0.096; -0.015)
EQ-5D-5L 3 months	0.837 (*n* = 168)	0.182	0.795 (*n* = 244)	0.218	-0.041 (-0.082; -0.001)
EQ-5D-5L 6 months	0.834 (*n* = 170)	0.183	0.789 (*n* = 242)	0·211	-0.045 (-0.084; -0.005)
EQ-5D-5L 9 months	0.838 (*n* = 166)	0.173	0.794 (*n* = 233)	0.207	-0.044 (-0.082; -0.005)
EQ-5D-5L 12 months	0.831 (*n* = 163)	0.196	0.780 (*n* = 235)	0.216	-0.051 (-0.092; -0.009)
**QALYs at 12 months**	0.833 (*n* = 160)	0.155	0.789 (*n* = 230)	0.188	-0.045 (-0.080; -0.009)
**EQ-5D VAS**					
EQ-5D VAS Baseline	80.66 (*n* = 171)	14.05	76.39 (*n* = 244)	18.32	-4.27 (-7.54; -0.99)
EQ-5D VAS 3 months	80.77 (*n* = 168)	15.65	74.23 (*n* = 244)	20.15	-6.53 (-10.17; -2.90)
EQ-5D VAS 6 months	80.11 (*n* = 170)	16.44	75.26 (*n* = 243)	18.50	-4.85 (-8.32; -1.37)
EQ-5D VAS 9 months	80.07 (*n* = 166)	15.31	75.93 (*n* = 234)	18.29	-4.13 (-7.55; -0.72)
EQ-5D VAS 12 months	79.41 (*n* = 162)	16.04	74.69 (*n* = 235)	19.47	-4.73 (-8.37; -1.09)

### Primary analysis

The results of the incremental analysis are presented in Table 5. In the base case cost-utility analysis, which used the imputed data of the PP population and adjusted for baseline covariates, Mixed-use was found to be £1,348.82 cheaper per participant than Single-use, with no significant difference in QALYs. At a willingness-to-pay threshold of £20,000, Mixed-use was cost-effective in 99.56% of the 10,000 simulations.

### Secondary analyses

The findings of all sensitivity and subgroup analyses are presented in Table 5. The findings for the PP population were robust across the mITT and ITT populations. However, the ITT population had a greater difference in costs between groups and a higher net monetary benefit. This difference was driven by the imputation model predicting that those with missing data in the Single-use group would incur substantially higher visit costs, and thus total costs, relative to the Mixed-use group. Therefore, while the imputed ITT model increased costs in both groups, the relative difference was greater in the Single-use group, making Mixed-use appear more cost-effective. An overview of imputed cost values per cost category and population can be found in Supplementary Table 4.

The findings in all subgroup analyses, the complete case analysis, and the analysis including participants’ personal expenses were all consistent with the base-case analysis. The threshold analysis found that, in the PP population, the price of single-use catheters would have to decrease by more than 74% for the Single-use strategy to become the cost-effective option. This would equate to a price of £0.44 per catheter.

**Table 5:** Results of incremental analysis

	Incremental Costs Mean (95% CI)	Incremental QALYs Mean (95% CI)	NMB (£20,000)	CEAC (£20,000)	NMB (£30,000)	CEAC (£30,000)
PP (Imputed sample, adjusted)	-£1348.82; [-1939.98; -757.65]	-0.001; [-0.026; 0.024]	£1328.82	99.56%	£1318.82	98.8%
PP (complete cases, adjusted)	-£1309.57 [-2201.11; -418.02]	0.003 [-0.036; 0.041]	£1369.57	97.9%	£1399.57	95.6%
PP (Imputed sample, unadjusted)	-£1432.10 [-1886.70; -977.49]	0.044 [0.011; 0.078]	£2312.10	100%	£2752.10	100%
PP (Imputed sample, adjusted, including out-of-pocket expenses)	-£1640.94 [-2426.60, -855.29]	-0.001 [-0.026; 0.023]	£1620.94	99.6%	£1610.94	99.2%
ITT (imputed, adjusted)	-£1900.00; [-2248.87; -1551.13]	0.003 [-0.012; 0.020]	£1960.00	100%	£1990.00	100%
mITT (imputed, adjusted)	-£1303.49; [-1806.40; -800.58]	-0.0004; [-0.020; 0.019]	£1295.49	100%	£1291.49	100%
mITT (female subgroup, adjusted, imputed)	-£1341.54 [-2208.80; -474.28]	0.009 [-0.032; 0.051]	£1521.54	99.3%	£1341.54	98.3%
mITT (male subgroup, adjusted, imputed)	-£1364.68 [-2013.18; -716.19]	-0.008 [-0.032; 0.017]	£1204.68	99.8%	£1124.68	97.8%
mITT (low Barthel subgroup, adjusted, imputed)	-£2284.99 [-4051.80; -518.17]	0.014 [-0.072; 0.099]	£2564.99	96.6%	£2704.99	94.4%
mITT (high Barthel subgroup, adjusted, imputed)	-£1230.45 [-1773.79; -687.11]	0.000 [-0.022; 0.021]	£1230.45	100%	£1230.45	100%

## Discussion

This study found that the Mixed-use strategy with both reusable and single-use catheters is cost-effective when compared to the Single-use only, due primarily to the reduction in catheter costs with no significant difference in QALYs. These findings were robust across all analyses, with the intervention found to be cost-effective between 96.6% and 100% of the time at a willingness-to-pay threshold of £20,000 per QALY. These findings may be an underestimate of the full societal benefit, as the expected reduction in environmental costs associated with reuse were not included [24].

These findings are consistent with those of the 2013 decision analytic model [11]. However, their study adopted a lifetime horizon and focussed predominantly on patients with spinal cord injury. Our findings expand on this by providing evidence of reusable catheters being cost-effective for a more diverse patient population and in the short-term. Our findings also contradict IC policies that justify reimbursing single-use catheters instead of reusable catheters based on cost savings from fewer UTIs. A 2024 paper by Zhao et al. critiqued one such policy in the US by stating that, in addition to no evidence of increased UTI risk, these policies did not adequately account for the substantial costs associated with using more single-use catheters [25]. Our findings support Zhao’s position, but we argue that offering patients the flexibility to use both single-use and reusable catheters can still be cost-effective while also championing patient choice.

Further, qualitative interviews with MultICath participants showed that many IC users worry about the economic and environmental impact of their catheter use [6], and preference data gathered during the trial showed that 93.2% to 95.9% of Mixed-use participants thought the NHS should offer both single and reusable catheters (Supplementary Table 5). Our findings support increasing patient choice in line with these concerns and preferences.

A strength of this study is that it was conducted alongside an RCT and was, therefore, able to leverage the 12-month patient-level data. This is a widely accepted form of economic evaluation [26]. However, MultICath did have a significant number of withdrawals, particularly in the intervention group. It is reassuring that the findings of the ITT analysis with imputed data were broadly consistent with the base-case PP analysis. However, this analysis was conducted under the assumption of missing at random, which may not be realistic due to factors associated with withdrawals related to the intervention. This might explain why the imputed ITT model predicted substantially higher visit costs for the Single-use group, leading to a higher NMB, than the PP or mITT models, as the factors contributing to withdrawal in the Single-use group might not have been fully captured by our model. It is important to note that the purpose of this study was to assess if there is economic evidence to provide patients with the choice of a reusable catheter, as opposed to changing the standard of care. Therefore, while the limitations of our handling of missing data should be acknowledged, it is unlikely to majorly impact our conclusions.

Additionally, the resource use form used in this study was not specific to the intervention, except for UTI antibiotic use. While this is an accepted form of resource use collection for economic evaluation, it did not allow us to analyse the impact of the intervention on direct care use, except for antibiotics [27]. All resource and catheter use data was self-reported by patients, which could have led to recall bias. While it is possible that Mixed-use patients may have felt pressure to over-report their reuse, this is unlikely because they were explicitly given the freedom to use single-use and reusable catheters in the ratio of their choosing.

Lastly, as the intervention is not currently covered by the NHS, the intervention was assumed to equal the commercial list price, and the cost of single-use catheters to equal the mean cost of Nelaton catheters on the PCA. In practice, if covered, the NHS would probably negotiate a lower price for the intervention than the commercial price, increasing the cost-savings. However, the magnitude of these cost savings would depend on the price of the specific single-use catheter that someone was switching from. Uncertainty around the cost of catheters was evaluated through a threshold analysis, with findings supporting Mixed-use until single-use catheters cost less than £0.44.

Our study found economic evidence to support offering reusable catheters in the NHS. These findings may also apply to other countries with high single-use catheter costs, and future work should be done into generating the evidence necessary for provision or reimbursement in these contexts. Future research could also expand on these findings by incorporating the environmental cost savings associated with the reduction in plastic waste into the model. Additionally, a decision analytic model could provide valuable insight into the longer-term cost implications of adopting a Mixed-use strategy.

In conclusion, this study found robust evidence for the cost-effectiveness of Mixed-use IC strategies and supports the provision of reusable catheters in the NHS and potentially other health services. The provision of reusable catheters would reduce costs and enhance patient choice with no HRQoL detriment, benefiting both patients and the health service.

## Electronic Supplementary Material

Below is the link to the electronic supplementary material.

Supplementary Material 1

## Data Availability

Consent was not obtained from participants for data sharing. Authors will consider reasonable request to make relevant anonymised participant level data available.

## References

[1] Cardozo L, Rovner E, Wagg A, Wein A, Abrams P. Incontinence. 7th ed. Bristol: ICI-ICS. International Continence Society; 2023.

[2] Berendsen SA, van Doorn T, Blok BFM. Trends in the use and costs of intermittent urinary catheters in the Netherlands from 1997 to 2018: A population-based observational study. Neurourol Urodyn. 2021;40:876–82. 10.1002/nau.24643. 33645866 10.1002/nau.24643 PMC8049077

[3] Lapides J, Diokno A, Silber S, Lowe B. Clean, intermittent self-catheterization in the treatment of urinary tract disease. Hournal Urol. 1972;107:458–61. 10.1016/s0022-5347(17)61055-3 5010715

[4] McClurg D, Coyle J, Long A, Moore K, Cottenden A, May C, Fader M. A two phased study health care professionals’ perceptions of single or multi-use of intermittent catheters. Int J Nurs Stud. 2017;72. 10.1016/j.ijnurstu.2017.04.009. 10.1016/j.ijnurstu.2017.04.009 28505559

[5] National Institute for Health and Care Excellence (NICE). Late-stage assessment final scope: GID-HTE10049 intermittent urethral catheters for long-term urinary management in adults. 2024.

[6] Clancy B, Murphy C, Avery M, Macaulay M, May C, Fader M. Reusable Intermittent Catheters are Acceptable but Product Innovation is Needed. J Wound Ostomy Cont Nurs. 2025;52:59–65. 10.1097/WON.0000000000001141. 10.1097/WON.0000000000001141 39836002

[7] Murrin S. Reducing Medicare’s payment rates for intermittent urinary catheters can save the program and beneficiaries millions of dollars each year. 2022.

[8] Sun AJ, Comiter CV, Elliott CS. The cost of a catheter: An environmental perspective on single use clean intermittent catheterization. Neurourol Urodyn. 2018;37:2204–8. 10.1002/nau.23562. 30106190 10.1002/nau.23562

[9] Prieto JA, Murphy CL, Stewart F, Fader M. Intermittent catheter techniques, strategies and designs for managing long-term bladder conditions. Cochrane Database Syst Reviews 2023. 2021. 10.1002/14651858.CD006008.pub5. 10.1002/14651858.CD006008.pub5 PMC8547544 34699062

[10] Fader M, Macaulay M, Wilson N, Chadwick T. Reusable catheters for intermittent catheterisation: a randomised, open-label, non-inferiority trial comparing combined reusable and single-use catheters (Mixed-use) with single-use catheters. Int J Nurs Stud. 2026.

[11] Bermingham SL, Hodgkinson S, Wright S, Hayter E, Spinks J, Pellowe C. Intermittent self catheterisation with hydrophilic, gel reservoir, and non-coated catheters: a systematic review and cost effectiveness analysis. BMJ. 2013;346:e8639–8639. 10.1136/bmj.e8639. 23303886 10.1136/bmj.e8639 PMC3541473

[12] Fader M, Macaulay M, Wilson N, Goudie N, Chadwick TJ, Abouhajar A, Jones J, Watson G, Avery MR, Broadbridge J, Buckley BS, Clancy B, Cottenden A, Dickson S, Guerrero K, Hagen S, James CP, Khasriya R, Little P, May CR, McClurg D, Moore M, Murphy C, Prieto J, Reading I, Shields C, Timoney A, Wilks SA. Trial to compare mixed-use (multi-use and single-use) intermittent catheter management with single-use management over 12 months (The MultICath Trial): protocol for a non-inferiority randomised controlled trial. BMJ Open. 2024;14:e088483. 10.1136/bmjopen-2024-088483. 39645276 10.1136/bmjopen-2024-088483 PMC11367288

[13] Wilks S, Macaulay M, Prieto J, Avery M, Bryant C, Delgado D, Murphy C, Morris N, Fader M. How to clean a catheter: development of an intervention for intermittent catheter reuse. BJUI Compass. 2025;6. 10.1002/bco2.487. 10.1002/bco2.487 PMC11794242 39917585

[14] NHS Business Service Authority. Prescription cost analysis – England 2023/24. 2024. https://www.nhsbsa.nhs.uk/statistical-collections/prescription-cost-analysis-england/prescription-cost-analysis-england-202324. Accessed 8 May 2025.

[15] Office for National Statistics. Consumer price inflation tables. 2025. https://www.ons.gov.uk/economy/inflationandpriceindices/datasets/consumerpriceinflation. Accessed 8 May 2025.

[16] NHS England. National cost collection data publication: National Schedule 2023/24. 2024. https://app.powerbi.com/view?r=eyJrIjoiZGQxYjNkOGUtOTIwMC00N2VjLWEyM2EtYjAzOGMwNWU5ODQ1IiwidCI6IjM3YzM1NGIyLTg1YjAtNDdmNS1iMjIyLTA3YjQ4ZDc3NGVlMyJ9. Accessed 8 May 2025.

[17] Jones KC, Weatherly H, Birch S, Castelli A, Chalkley M, Dargan A, Findlay D, Gao M, Hinde S, Markham S, Smith D, Teo H. Unit costs of health and social care 2024 manual. Kent (UK). 2025.

[18] NHS Business Services Authority. NHS Electronic Drug Tariff. 2024.

[19] Herdman M, Gudex C, Lloyd A, Janssen MF, Kind P, Parkin D, Bonsel G, Badia X. Development and preliminary testing of the new five-level version of EQ-5D (EQ-5D-5L). Qual Life Res. 2011;20:1727–36. 10.1007/s11136-011-9903-x. 21479777 10.1007/s11136-011-9903-x PMC3220807

[20] Hernández Alava M, Pudney S, Wailoo A. Estimating the Relationship Between EQ-5D-5L and EQ-5D-3L: Results from a UK Population Study. PharmacoEconomics. 2023;41:199–207. 10.1007/s40273-022-01218-7. 36449173 10.1007/s40273-022-01218-7 PMC9883358

[21] Manca A, Hawkins N, Sculpher MJ. Estimating mean QALYs in trial-based cost‐effectiveness analysis: the importance of controlling for baseline utility. Health Econ. 2005;14:487–96. 10.1002/hec.944. 15497198 10.1002/hec.944

[22] Willan AR, Briggs AH, Hoch JS. Regression methods for covariate adjustment and subgroup analysis for non-censored cost‐effectiveness data. Health Econ. 2004;13:461–75. 10.1002/hec.843. 15127426 10.1002/hec.843

[23] Faria R, Gomes M, Epstein D, White IR. A guide to handling missing data in cost-effectiveness analysis conducted within randomised controlled trials. Pharmacoeconomics. 2014;32:1157–70. 10.1007/s40273-014-0193-3 10.1007/s40273-014-0193-3 PMC4244574 25069632

[24] Keil M, Viere T, Helms K, Rogowski W. The impact of switching from single-use to reusable healthcare products: a transparency checklist and systematic review of life-cycle assessments. Eur J Public Health. 2023;33:56–63. 10.1093/eurpub/ckac174. 36433787 10.1093/eurpub/ckac174 PMC9898010

[25] Zhao CC, Comiter CV, Elliott CS. Perspectives on technology: Single-use catheters – evidence and environmental impact. BJU Int. 2024;133:638–45. 10.1111/bju.16313. 38438065 10.1111/bju.16313

[26] Drummond M, Sculpher M, Claxton K, Stoddart G, Torrance G. Methods for the economic evaluation of health care programmes. 4th ed. Oxford: Oxford University Press; 2015.

[27] Janssen LMM, Drost RMWA, Paulus ATG, Garfield K, Hollingworth W, Noble S, Thorn JC, Pokhilenko I, Evers SMAA. Aspects and challenges of resource use measurement in health economics: towards a comprehensive measurement framework. Pharmacoeconomics. 2021;39. 10.1007/s40273-021-01048-z PMC8352823 34169466

