# Supplementary material for: An economic evaluation of the MultICath randomised controlled trial, comparing combined use of reusable and single-use intermittent catheters to single-use catheters only
Source: Cost Eff Resour Alloc. 2026 May 9;24:65. doi: 10.1186/s12962-026-00750-z (PMC13198048; doi:10.1186/s12962-026-00750-z)
Supplement: Supplementary file 1 — Supplementary Material 1 [file 12962_2026_750_MOESM1_ESM.docx]

**Supplementary material for “An economic evaluation of the MultICath Randomised Controlled Trial, comparing combined use of reusable and single-use intermittent catheters to single-use catheters only”, Sara McCloskey, Tracey H Sach, Margaret Macaulay, Miriam R Avery, Thomas J Chadwick, Bridget Clancy, Sylvia Dickson, Nicola Goudie, Karen Guerrero, Suzanne Hagen, Cathy Murphy, Gillian C Watson, Nina Wilson, Ruth Wood, Mandy Fader. Published in Cost Effectiveness and Resource Allocation.**

**Corresponding author: Professor Tracey Sach, University of Southampton, t.sach@soton.ac.uk**

**Supplementary figure 1**: Per Protocol (PP) cost-effectiveness acceptability curve

**Supplementary figure 2**: Per protocol incremental cost-effectiveness plane

**Supplementary table 1**: Intention to Treat (ITT) resource use

|  | **Mixed-use strategy (n=289)** | | **Single-use catheters only strategy (n=289)** | | **Mean difference** |
| --- | --- | --- | --- | --- | --- |
|  | **Mean** | **Std dev** | **Mean** | **Std dev** | **(95% CI)** |
| **Intervention (number)** |  |  |  |  |  |
| Cliny catheters | 8.82  (n=192) | 5.30 | 0 | 0 | 8.82 (8.21; 9.43) |
| Cliny kits | 3.40  (n-192) | 4.00 | 0 | 0 | 3.40 (2.93; 3.86) |
| Single-use catheters | 839.88  (n=186) | 1294.47 | 1527.62  (n=224) | 783.23 | -687.73 (-891.80; -483.66) |
| **Primary and secondary care (number)** |  |  |  |  |  |
| GP visits | 2.16  (n=196) | 2.89 | 2.95  (n=235) | 4.27 | -0.79 (-1.50; -0.08) |
| Nurse visits | 2.18  (n=196) | 2.70 | 3.05  (n=235) | 7.82 | -0.86 (-2.01; 0.29) |
| Outpatient visit | 2.91  (n=196) | 5.62 | 3.20  (n=235) | 4.99 | -0.28 (-1.29; 0.72) |
| Inpatient visit | 0.23  (n=201) | 0.63 | 0.27  (n=237) | 0.77 | -0.05 (-0.18; 0.09) |
| A&E visit | 0.18  (n=196) | 0.49 | 0.26  (n=235) | 0.79 | -0.09 (-0.21; 0.04) |
| Other visits | 0.02  (n=196) | 0.20 | 0.04 | 0.59 | -0.02 (-0.10; 0.07) |
| **Medication- number of people who took at least one antibiotic** |  |  |  |  |  |
| Medication – all antibiotics for UTI treatment | 70  (n=178) | 87.19 | 106  (n=215) | 107.74 | -36 (-55.72; -16.28) |
| **Participant personal costs** |  |  |  |  |  |
| Participant resource use e.g. private appointments, over the counter purchases | 90  (n=197) | 98.38 | 107  (n=235) | 117.28 | -17 (-37.71; 3.71) |

Note: ITT and mITT available cases were the same

**Supplementary table 2**: ITT unadjusted costs

|  | **Mixed-use strategy (n=289)** | | **Single-use catheters only strategy (n=289)** | | **Mean difference** |
| --- | --- | --- | --- | --- | --- |
|  | **Mean** | **Std dev** | **Mean** | **Std dev** | **(95% CI)  £’s** |
| **Intervention** | | | | |  |
| Multiuse catheters and accessories (kits, single catheters, Optilube, and Milton, container, and syringe) | £156.00 (n=192) | £85.92 | 0 | 0 | £156.00  (£145.69; £166.31) |
| Single-use catheters | £1419.40  (n=186) | £2187.66 | £2581.67  (n=224) | £1323.66 | -£1162.27  (-£1507.14; -£817.39) |
| **Primary and secondary care** |  |  |  |  |  |
| GP visits | £80.04  (n=196) | £158.15 | £109.811  (n=235) | £107.07 | -£29.07  (-55.18; -2.96) |
| Nurse visits | £17.14  (n= 196) | £21.14 | £23.86  (n=235) | £61.26 | -£6.72  (-15.74; 2.30) |
| Hospital consultant visit (remote and in person) | £467.37  (n=196) | £899.43 | £507.68  (n=235) | £798.99 | -£40.32  (-201.19; 120.56) |
| Inpatient visit | £588.73  (n=202) | £1597.77 | £683.23  (n=238) | £1892.72 | -£94.50  (-426.08; 237.08) |
| A&E visit | £48.75  (n=196) | £133.67 | £72.03  (n=235) | £216.85 | -£23.28  (-£58.22; £11.66) |
| Other visits | £35.56  (n=197) | £105.48 | £52.68  (n=235) | £287.31 | -£17.12  (-59.57; 25.33) |
| **Medication** |  |  |  |  |  |
| Medication – All antibiotics for UTI treatment | £4.94 (n=178) | £26.06 | £8.15  (n=215) | £19.83 | -£3.21  ( -7.76; 1.35) |
| **Participant personal costs** |  |  |  |  |  |
| Participant resource use e.g. private appointments, over the counter purchases | £359.72  (n=197) | 1638.65 | £691.21  (n=235) | 3450.871 | -£331.49  (-858.50; 195.51) |
| **Total health care costs** | £2468.79 (n=173) | 2109.48 | £3954.05  (n=204) | 2629.39 | -£1485.49  (-£1974.01; -996.51) |

Note: ITT and mITT available cases were the same

**Supplementary table 3**: ITT EQ-5D

|  | **Mixed-use strategy (n=289)** | | **Single-use catheters only strategy (n=289)** | | **Mean difference** |
| --- | --- | --- | --- | --- | --- |
|  | **Mean** | **Std dev** | **Mean** | **Std dev** | **(95% CI)** |
| **EQ-5D-5L Index** |  |  |  |  |  |
| EQ-5D-5L Baseline | 0.84 (n=207) | 0.18 | 0.77 (n=244) | 0.22 | -0.07  (-0.1; -0.03) |
| EQ-5D-5L 3 months | 0.85 (n=204) | 0.18 | 0.80 (n=244) | 0.22 | -0.05  (-0.09; -0.01) |
| EQ-5D-5L 6 months | 0.84 (n=206) | 0.19 | 0.79 (n=242) | 0.21 | -0.05  (-0.09; -0.01) |
| EQ-5D-5L 9 months | 0.84 (n=200) | 0.17 | 0.79 (n=233) | 0.21 | -0.05  (-0.09; -0.01) |
| EQ-5D-5L 12 months | 0.83 (n=197) | 0.19 | 0.78 (n=235) | 0.22 | -0.05  (-0.09; -0.01) |
| **QALYs at 12 months** | 0.84 (n=193) | 0.15 | 0.79 (n=230) | 0.19 | -0.05  (-0.08; -0.02) |
| **EQ-5D VAS** |  |  |  |  |  |
| EQ-5D VAS Baseline | 81.4 (n=207) | 14.04 | 76.39 (n=244) | 18.32 | -5.01  (-8.07; -1.94) |
| EQ-5D VAS 3 months | 81.09 (n=204) | 15.51 | 74.23 (n=244) | 20.15 | -6.86  (-10.25; -3.47) |
| EQ-5D VAS 6 months | 80.41 (n=206) | 16.71 | 75.26 (n=243) | 18.50 | -5.15  (-8.44; -1.86) |
| EQ-5D VAS 9 months | 80.32 (n=200) | 15.64 | 75.93 (n=234) | 18.29 | -4.38  (-7.62; -1.14) |
| EQ-5D VAS 12 months | 79.47 (n=196) | 16.45 | 74.69 (n=235) | 19.47 | -4.78  (-8.23; -1.33) |

Note: ITT and mITT available cases were the same

**Supplementary table 4**: Mean (SD) imputed costs per category and population

|  | **PP** | **mITT** | **ITT** |
| --- | --- | --- | --- |
| **Catheter costs** | Single use:  £2,535.67 (1,330.89)  Mixed-use:  £1,307.44 (1,087.37) | Single use:  £2,541.25 (1,334.13)  Mixed-use:  £1,511.75 (1,178.83) | Single use:  £2,456.68 (1,349.03)  Mixed-use:  £1,684.80 (1,273.36) |
| **Visit costs** | Single use:  £1,380.87 (2,307.96)  Mixed-use:  £1,179.88 (1,891.36) | Single use:  £1,396.69 (2,321.28)  Mixed-use:  £1,136.73 (1,862.64) | Single use:  £1,959.62 (3,157.64)  Mixed-use:  £1,166.19 (1,885.37) |
| **Antibiotic costs** | Single use:  £8.54 (19.88)  Mixed-use:  £5.66 (30.41) | Single use:  £8.46 (19.76)  Mixed-use:  £6.15 (31.20) | Single use:  £9.71 (20.48)  Mixed-use:  £8.67 (38.46) |

**Supplementary table 5**: Catheter management preferences in Mixed-use group.

| **Question** | **6 months (n=207)** | **12 months (n=197)** |
| --- | --- | --- |
| **Which of the following catheters do you prefer to use? n (%)** |  |  |
| Single-use catheters only | 22 (10.7%) | 32 (16.2%) |
| Mostly single-use catheters with occasional use of multi-use catheters | 35 (17.0%) | 29 (14.7%) |
| Regular use of both single-use and multi-use catheters | 68 (33.0%) | 64 (32.5%) |
| Mostly multi-use catheters with occasional use of single-use catheters | 73 (35.4%) | 68 (34.5%) |
| Multi-use catheters only | 6 (2.9%) | 3 (1.5%) |
| Something else | 2* (1.0%) | 1^#^ (0.5%) |
| n | 206 | 197 |
| **Do you think the NHS should provide, n(%)** |  |  |
| Single use catheters only? | 7 (3.4%) | 7 (3.6%) |
| Multi-use catheters only? | 7 (3.4%) | 1 (0.5%) |
| Both single-use and Multi-use catheters? | 191 (93.2%) | 188 (95.9%) |
| n | 205 | 196 |
| **How important is this reduced catheter wastage to you? n(%)** |  |  |
| Very important | 102 (49.5%) | 100 (50.8%) |
| Quite important | 86 (41.7%) | 84 (42.6%) |
| Not important | 18 (8.7%) | 13 (6.6%) |
| n | 206 | 197 |

* The two reasons were – (1) Away from home - single use only; At home- mostly multi use

 (2) Quote   If the design of the multi-use catheter was the same as the single use one I would like to use it

# Reason not given

**Supplementary table 6**: HEAP

**MultICath Study Health Economics Analysis Plan (HEAP) v2.1** (Reference: Thorn, J. C., et al. 2021. Content of Health Economics Analysis Plans (HEAPs) for Trial-Based Economic Evaluations: Expert Delphi Consensus Survey. *Value in Health,* 24, 539-547.)

| **Section 1: Administrative information** | | | | | | | | |  |
| --- | --- | --- | --- | --- | --- | --- | --- | --- | --- |
| 1.1 | Title | | | Health economics analysis plan for the MultICath Study: A noninferiority randomised controlled trial to compare mixed  (multi/single-use) catheter management with single-use catheter management by intermittent catheter users over 12 months | | | | |  |
| 1.2 | Trial registration number | | | ISRCTN68472863 (ISRCTN registry) | | | | |  |
| 1.3 | Source of funding | | | National Institute for Health Research, PGfAR (Programme Grants for Applied Research) – RP-PG-0610-10078 | | | | |  |
| 1.4 | Purpose of HEAP | | | The purpose of this HEAP is to describe the analysis and reporting procedure intended for the economic analyses to be undertaken. The analysis plan is designed to ensure that there is no conflict with the protocol and associated statistical analysis plan and it should be read in conjunction with them. | | | | |  |
| 1.5 | Trial protocol version | | | This document has been written based on information contained in the trial protocol version 17.0, dated 05 July 2024 | | | | |  |
| 1.6 | Trial Statistical Analysis Plan (SAP) version | | | SAP Version: *2.0, Date: 12/07/2024* | | | | |  |
| 1.7 | Trial HEAP version | | | HEAP Version: 2.0, Date: 29/10/2024 | | | | |  |
| 1.8 | HEAP revisions | | | Date | HEAP Version | Protocol version and date | Section number | Reason for change/ amendment (include individual making change) |  |
|  |  |  |  | 15/7/2023 | 1 | 14.02, 07  December 2022 | NA | Baseline controlled version of HEAP. |  |
|  |  |  |  | 29/10/2024 | 2 | 17.0, dated 05 July 2024 | 5.11 | Sections on analysis were updated to reflect a focus on estimation rather than specifying a priori acceptable differences in costs and outcomes |  |
|  |  |  |  | 4/12/2014 | 2.1 | 17.0, dated 05 July 2024 | Table 1b | Table added to make it clear the source data for each item |  |
| 1.9 | Roles and responsibilities | | | Version 1.0 of the HEAP was prepared by Dr Jeremy Jones. In September 2024 Professor Tracey Sach took over as the senior health economist and revised the HEAP in light of the latest protocol, SAP and wider literature around methods. Sara McCloskey joined as the trial health economist in January 2025. Sara McCloskey will conduct and report the economic evaluation in accordance with the HEAP and under Tracey Sach’s supervision. | | | | |  |
| 1.10a | Signature of Tracey Sach, Senior health economist Date: | | | 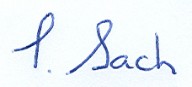    4/12/24 | | | | |  |
| 1.10b | Signature of Thomas Chadwick, Senior trial statistician | | | 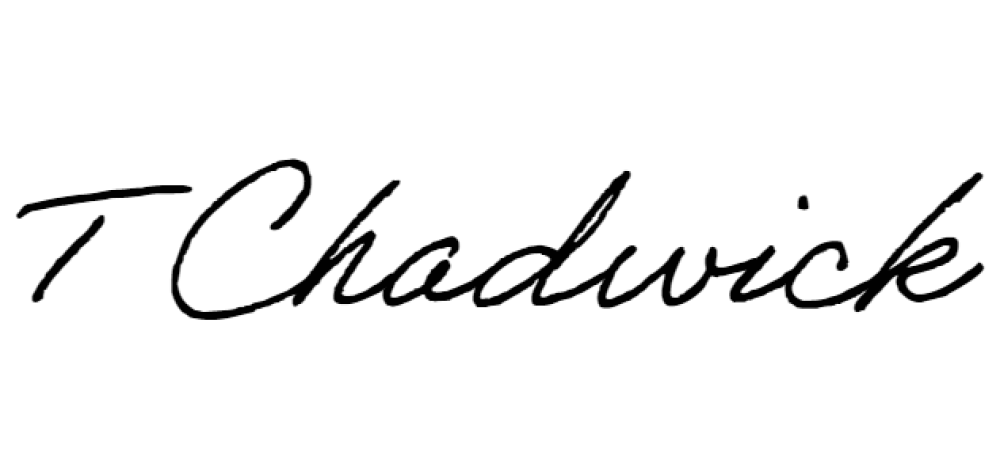 | | | | |  |
|  | Date: 07/01/2025 |  | | | | | | | |
| 1.10c | Signature of Mandy  Fader, Chief Investigator  Date: 07.01.25 | 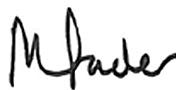 | | | | | | | |
| **Section 2: Trial introduction & background** | | | | | | | | | |
| 2.1 | Trial background and rationale | *Information should be read in line with the protocol.*    If the bladder is not completely emptied it can cause incontinence, repeat urinary tract infections (UTI) and other symptoms and is a common urological issue. Where there is no underlying treatable cause for the retention of urine in the bladder the standard management approach is to teach the individual or carer clean intermittent catheterisation (IC). IC is a process where a catheter (tube) is passed into the bladder to drain urine and then removed. This process can be needed 4 to 5 times a day.    Catheters were used multiple times by an individual user when IC was first introduced because it was believed the main source of any potential infection was a person’s own peri-urethral flora. However, in the 1980s single-use, hydrophilic coated catheters became available on NHS prescription in the UK and as such single use catheters became more common than reusable catheters. A trend which was also facilitated by the labelling change of catheters by the Medicines and Healthcare products Regulatory Agency (MHRA) that stipulated that products and devices could not be reused unless the manufacturer provided validated reprocessing information.    No evidence to support any difference in incidence of UTI between use of an aseptic or clean technique of insertion, between coated or uncoated catheters or between single (sterile) and multiple-use (clean) catheters was found in a Cochrane review (Prieto JA et al 2021). However, IC users have indicated that they find carrying multiple packets of catheters cumbersome and less discreet than a multi-use catheter. However, there have also been reports that it is hard to dispose of packaging and components for single use catheters in public toilets.    In trial development the IC user group indicated that whilst there is advantages and disadvantages to multi-use compared with single use catheters most would not wish to change to using multi-use catheters exclusively. A mixed package combining single and multi-use catheters was preferred and therefor this trial offered a mixed package as the most likely future alternative strategy for IC. | | | | | | | |
| 2.2 | Aim(s) of the trial | The MultICath study’s primary aim is to establish whether a strategy of intermittent urinary catheter mixed use is no worse than a strategy of intermittent urinary catheter single (sterile) use only in terms of incidence of microbiologically-confirmed symptomatic and help-seeking/self-help UTI over 12 months (post-two-week learning period) using a randomised controlled trial design.    The secondary aims are:  - to establish whether a strategy of intermittent urinary catheter mixed use is no worse than a strategy of intermittent urinary catheter single (sterile) use only in terms of incidence of microbiologically-confirmed symptomatic and help-seeking/selfhelp UTI over 6 months (post-two-week learning period) using a randomised controlled trial design.    - to assess non-microbiologically confirmed UTI, haematuria, user acceptability, user preference and cost of providing the mixed package and associated costs. | | | | | | | |
| 2.3 | Objectives and/or  research hypotheses of the trial | Primary objective: to determine if a mixed-use strategy is no worse than a single-(sterile)-use-only strategy for incidence of microbiologically-confirmed symptomatic and help-seeking UTI over 12 months (post two week learning period) in people practicing intermittent urinary catheterisation.    Secondary objectives: to determine if a mixed-use strategy is no worse than a single-(sterile)-use-only strategy for incidence of microbiologically-confirmed symptomatic and help-seeking UTI over 6 months (post two week learning period) in people practicing intermittent urinary catheterisation, to determine if a mixed-use strategy is no worse than a single-use only strategy for outcomes of urinary tract infection (non-microbiologically confirmed), haematuria, user acceptability, user preference and cost for the NHS. | | | | | | | |
| 2.4 | Trial population | **Inclusion criteria:** Adult men and women aged ≥ 18 years, currently using or preparing to start IC )via the urethra), performed by self or sole carer. Patients whi have been IC users for at least ≥ six weeks, where IC planned to continue for >12 months and two weeks. Able and willing to adhere to a 12‐month follow up period and has provided written informed consent for participation in the trial prior to any trial specific procedures    **Exclusion criteria**: Age < 18 years. Use of IC for self- dilatation of urethral stricture without bladder drainage. Non-urethral route for catheterisation e.g. Mitrofanoff. External, non-sole carer required for IC (i.e. where sterile technique and catheter is required e.g. visiting community nurse performs IC). Inability to give informed consent or have primary outcome information collected. Employee or relation of employee of a manufacturer or distributor of IC catheters. Women who report they are pregnant or who plan to become pregnant during the trial. Participation in another trial. Patients in the terminal stages of an illness. Anything that, in the opinion of the Chief Investigator or a delegated member of the research team, prohibits the patient’s participation in the trial | | | | | | | |
| 2.5 | Intervention(s) and comparator(s) | **Intervention**: the trial intervention consists of a mixed strategy, using both multi- and single-use catheters. This was adopted following feedback from the user group indicated that a complete change to multi-use catheters would not be preferred by most people using IC and therefore would not reflect the likely future use of this alternative strategy.  Implementation of this strategy takes place in two stages:  • 2-week learning period, during which participants initially use the trial catheter for single use, then progress to cleaning and re-using them. The aim of this period is to have participants re-using the catheters for at least half of their daily catheterisations;  • the trial phase, in which participants are encouraged to continue the proportion of re- use achieved during the learning period, although less frequent use is permitted. Guidelines on cleaning, storing and lubricating catheters will be provided to participants in booklet and video format – these were developed during an earlier phase of the MultICath programme, supplemented by a user evaluation conducted by the catheter manufacturer. During the trial phase participants will be provided with catheter kits (re-usable catheter, case and carrying pouch), additional re-usable catheters, lubricant along with supplies of cleaning and storing products.    The catheter to be used in the intervention arm is a Cliny silicone catheter which is CE-marked for repeated use over 28 days when cleaned with Milton® between uses. Cliny catheters are uncoated and therefore require lubrication for use (three types of waterbased to be used – K-Y, Sutherland or Optilube lubricating jelly.)    **Comparator**: Single-use catheter use only (management as usual). Participants will continue to use single-use catheters obtained by their usual method and will be required to stop using any multiuse catheters, if they are doing so. | | | | | | | |
| 2.6 | Trial design | | Non-inferiority, patient-randomised, controlled trial (RCT). Patients will be randomised in a 1:1 ratio to mixed catheter use (intervention) or single use catheter use (control).    Target recruitment is 578, allowing for 28% attrition (162) – expected completion per arm=208    Non-inferiority margin on outcome(s) is expressed as an odds ratio of 2.0 (see section 13.1 Sample Size Calculation (page 52-53) in the protocol. | | | | | | |
| 2.7 | Trial start and end dates | | Recruitment started in February 2021 (first patients recruited April 2021) and finished 12^th^ July 2024. The follow-up period is 12 months. | | | | | | |
| **Section 3: Economic approach/overview** | | | | | | | | | |
| 3.1 | Aim(s) of economic evaluation | | The aim of the economic evaluation is to address the question  “What is the cost-effectiveness of adopting a mixed strategy (combining multi- and single-use catheters) compared with singleuse catheter management by intermittent catheter users?”. | | | | | | |
| 3.2 | Objective(s) of economic evaluation | | The primary objective of the health economic evaluation is to estimate the cost-effectiveness of mixed catheter management versus single-use catheter management by intermittent catheter users in a within-trial economic evaluation, adopting a time horizon of twelve months. | | | | | | |

| 3.3 | | Overview of economic analysis | | | The economic analysis will be performed using individual patient level data from the trial and will take the form of a costconsequence and cost-utility analysis. Incremental costeffectiveness ratios will be calculated by taking a ratio of the difference in the mean costs and mean effects (expressed as Quality-Adjusted Life Years).    The analysis will focus on estimating cost and effect differences along with the probability that a mixed strategy is cost effective compared with single-use catheter management. (Whitehurst and Bryan, 2013). We do not specify a priori acceptable cost and effect differences as suggested by Bosmans et al (2008) because that is not the analyst’s role (Whitehurst and Bryan, 2013). |
| --- | --- | --- | --- | --- | --- |
| 3.4 | | Jurisdiction(s) | | | The trial is conducted in the UK which has a national health service (NHS), providing publicly funded healthcare, primarily free of charge at the point of use. |
| 3.5 | | Perspective(s) | | | The primary economic analysis will be from the NHS and personal social services (PSS) perspective. |
| 3.6 | | Time horizon(s) | | | The primary economic analysis will compare the costs and consequences of each arm over the trial follow-up period of twelve months from the end of the two-week learning period. |
| **Section 4: Economic data collection & management** | | | | | |
| 4.1 | | Statistical software | | | Excel will be used for exploratory analysis and Stata 18.5 SE for the main analysis. |
| 4.2 | | Identification of resources | | | Delivery of the intervention (including catheters, case, carrying pouch, soap, Milton© and lubricating jelly) will be measured to assess the cost of the intervention. In practice the training element would likely consist of an electronic booklet and video which would be freely distributed and so a cost will not be attached to this element of the intervention.    The impact on health care resource use that may differ between groups will be measured including primary (GP, nurse or other professional in surgery, over telephone or out of hours) and secondary care (inpatient admissions, outpatient appointments and A&E visits) resource use and to a limited extent personal expenditure on health care . |
| 4.3 | | Measurement of resource-use data | | | Resource-use data related to the intervention will be collected from the two-week learning period until 12 months using data collected in monthly questionnaires, completed by the researcher over the phone with the participant. Where this is missing data from the UTI questionnaire will be utilised where available.    Patient questionnaires completed with and by a researcher at 6 and 12 months follow up will be used to collect information on other primary and secondary care use.    See appendix 1 for copies of the monthly Catheter/UTI and 6/12 month health resource use questionnaires. |
| 4.4 | | Valuation of resourceuse data | | All resource use will be valued in monetary terms using appropriate UK unit costs or participant valuations estimated at the time of analysis.    Where available units for Primary Care Resources will be derived from the published Unit Costs of Health and Social Care Manual (Personal Social Services Research Unit, University of Kent) and Centre for Health Economics (University of York)(Jones et al, 2023).  Secondary care resource use unit costs will be derived from the NHS Reference Costs (NHS England 2023) and medication costs will be taken from the Prescription cost analysis (PCA)(NHSBSA 2023) or British National Formulary (BNF)(NICE, 2024). | |
| 4.5 | | Identification of outcome(s) | | The primary economic outcome measure will be Quality-Adjusted Life Years (QALYs) derived from utility scores, obtained using the EQ-5D-5L quality of life instrument (Herdman et al 2011). | |
| 4.6 | | Measurement of outcome(s) | | Measurements using EQ-5D-5L will be recorded at baseline, and at 3, 6, 9 and 12 months. Participants were given the EQ-5D-5L questionnaire in advance of a telephone interview during which the interviewer asked and recorded responses to the EQ-5D-5L. | |
| 4.7 | | Valuation of outcome(s) | | Utility scores will be derived from responses to the EQ-5D-5L. UK utility values will be derived using the approach recommended by NICE at time of the analysis. This is currently that published by Hernández Alava (2022, 2023).    These values will be used to estimate QALYs over the 12-month period (estimated using linear interpolation and area under the curve) adjusting for any imbalances in baseline EQ-5D-5L scores ( Manca et al 2005). | |
| **Section 5: Economic data analysis** | | | | | |
| 5.1 | | Analysis population | | Analyses will be undertaken per-protocol (PP) in line with the statistical analysis plan (SAP). The SAP defines the PP population as “**Per Protocol (PP):** this population contains all randomised patients who received their allocated intervention without major protocol violations*. For a patient to be included in the PP analysis population their percentage reuse must be at least 25% or an average of 0.75 per day during their potential treatment period with this period being of at least 6 months duration. The definition of how this will be calculated and implemented is described in 4.3 Treatment compliance.”    Further analyses may be undertaken, if considered informative – where there are substantial missing data the “complete case” method (Glick et al 2007) will be considered. See section 5.10 for preferred approach to accounting for missing data. | |
| 5.2 | | Timing of analyses | | The primary analysis will be a within-trial analysis conducted once all patients have been followed for twelve months (after completion of the two-week learning phase) and the database is locked. | |
| 5.3 | | Discount rates for costs and benefits | | Discounting will not be necessary as the primary analysis will be conducted with a twelve-month time horizon. | |
| 5.4 | | Cost-effectiveness threshold(s) | | The main primary analysis will be a cost utility analysis to estimate both the mean incremental cost and mean incremental effect (in terms of QALYs) of the mixed-strategy compared to single-use. The reported economic analysis will use a cost-effectiveness threshold (ʎ) of £20,000 (£30,000) per QALY (NICE, 2022). | |
| 5.5 | | Statistical decision rule(s) | | Mean differences in costs and QALYs between the treatment groups will be estimated with associated 95% confidence intervals. | |
| 5.6 | | Analysis of resource use | | Mean (sd) resource use per participant per resource will be estimated for each resource item and each randomised group. Mean difference (95% CI) in resource use per participant between groups (mixed-strategy vs single use) will be presented. See Appendix 2 for example result tables. | |
| 5.7 | | Analysis of costs | | Mean (sd) cost per participant will be estimated for each randomised group. Mean difference (95% CI) in cost per participant between groups (mixed-strategy vs single use) will be estimated. See Appendix 2 for example result tables. | |
| 5.8 | | Analysis of outcomes | | Mean (sd) utility and mean (sd) QALYS per participant per randomised group will be presented and mean difference (95% CI) in utility and QALYs between groups (mixed-strategy vs single use) will be estimated. See Appendix 2 for example result tables. | |
| 5.9 | | Data cleaning for analysis | | Before carrying out analyses, plausibility checks will be performed on the relevant data fields, such as resource use and reported outcome measures, including quality-of-life. Where problems are identified, the health economist will contact the data manager of the trial for clarification. | |
| 5.10 | | Missing data | | Trial data will be examined for any missing data. The level of missing data will be reported and the frequency and pattern of missing data will be examined, following the approach adopted by Faria et al. (2014) to missing data. This will include: the proportion of missing data by trial group at each follow-up period; missing data patterns to identify monotonic pattern or otherwise; association between missingness and baseline variables using logistic regression; and association between missingness and observed outcomes using logistic regression. This will inform the approach to dealing with missingness by assessing the likely mechanism of missingness. A logistic regression model will be used to identify variables predictive of missingness and these, along with covariates included in the final analysis, will be  included in the imputation model. Assuming the data is Missing at Random (MAR), the economic primary analysis will undertake multiple imputation using chained equations (MICE) to handle the missing cost and outcome data. The number of iterations of imputation will be based on the level of missing data with, for example, 30 repetitions for 30% missing data (minimum 10 imputations) (von Hippel 2007). | |
| 5.11 | | Analysis of cost-effectiveness | | Costs and outcomes will be presented descriptively as a cost consequence analysis, breaking down costs and outcomes for participants and carers along with sector/perspective (e.g. primary care, secondary care, participant).  A cost-utility analysis will be performed to estimate incremental cost and QALYs which will be combined to estimate an incremental cost-effectiveness ratio (ICER) from an NHS perspective, comparing mixed-use to single-use catheter strategy. A regression-based approach (such as seemingly unrelated regression equations, if appropriate) will be used in the primary cost-utility analysis (Willan et al 2004).  Both unadjusted and adjusted results will be presented. The adjusted analysis will be the primary analysis and will adjust for baseline cost, baseline utility, sex, age, medical condition, Barthel index, carer/no carer and antibiotic prophylaxis on the incidence of UTI in the two groups.  Note: the covariates were updated after the HEAP was signed to be consistent with the clinical paper and the available data. The revised covariate list is: baseline cost, baseline utility, sex, age, Barthel index, antibiotic prophylaxis, time in study, site, antibiotic use at baseline, and catheter use at baseline on the incremental costs and QALYs. | |
| 5.12 | Sampling uncertainty | | | The nonparametric bootstrapping approach will be used to determine the level of sampling uncertainty surrounding the mean ICERs by generating 10,000 estimates of incremental costs and benefits. Results of the bootstrap analysis will be presented on the cost-effectiveness plane. In addition, Cost-Effectiveness Acceptability Curves (CEACs) will be produced, which will show the probability of each randomised group being cost-effective for different values of willingness to pay. | |
| 5.13 | Subgroup analyses or  analysis of heterogeneity | | | Economic subgroup analysis choice will be driven by the subgroups included in the additional statistical analysis as outlined in Section 5.1 Analysis of primary outcome on page 36 of the SAP and the results of these. No subgroup analysis will be attempted with groups including fewer than 50 participants. | |
| 5.14 | Sensitivity analyses | | | Sensitivity analyses will be undertaken to explore key uncertainties around important parameters in the economic evaluation.   1. The impact of missing data will be explored by comparing primary analysis results using multiple imputation to a complete-case analysis if appropriate (See section 5.10) or by varying the time horizon of the analysis (i.e. taking a 6-month time horizon instead of 12 month). 2. Any sensitivity to protocol violations will be explored. 3. The base-case analysis will be repeated including participants’ personal costs if these prove significant. 4. The primary analysis will be repeated excluding outliers, if appropriate. | |
| **Section 6: Modelling – not proposed in the protocol** | | | | | |
| 6.1 | Extrapolation or decision analytic modelling | | | Decision analytic modelling to extrapolate costs and outcomes beyond the trial period will not be undertaken. | |
| 6.2 | Model type | | | N/A | |
| 6.3 | Model structure | | | N/A | |
| 6.4 | Treatment effect  beyond the end of the  trial | | | N/A | |
| 6.5 | Other key assumptions | | | N/A | |
| 6.6 | Methods for identifying and estimating parameters | | | N/A | |
| 6.7 | Model uncertainty | | | N/A | |
| 6.8 | Model validation | | | N/A | |
| 6.9 | Subgroup  analyses/heterogeneity | | | N/A | |
| **Section 7: Reporting/publishing** | | | | | |
| 7.1 | Reporting standards | | | Updated CHEERS guidelines (Husereau et al 2022) will be followed when reporting the health economic evaluation – see Appendix 3. | |
| 7.2 | Deviations from the HEAP | | | Any deviation from HEAP will be described and justified in the final published report. | |
| **Section 8: Appendices** | | | | | |
| 8.1 | Health economic collection tools | | Appendix 1 includes copies of the resource-use data collection sheets and resource-use questionnaires | | |

**HEAP Appendix 1 - Health economic data collection tools**

# Monthly Questionnaire – Control group

## Section A Urinary tract infection

Has participant had a UTI since the last contact? Yes □ No □

|  |
| --- |

If yes, please enter the total number of episodes and complete section A. If no, go to Section B.

| (Please tick ‘✓’ the appropriate answer) | **Yes** | | | | | **No** | | | | | **Don’t know** | | | | | |
| --- | --- | --- | --- | --- | --- | --- | --- | --- | --- | --- | --- | --- | --- | --- | --- | --- |
| **EPISODE 1** |  | | | | |  | | | | | |  | | | | |
| 1. Date symptoms started & ended |  |  |  |  |  | |  | **_** |  |  | | |  |  |  |  |
|  | **D** | **D** | **M** | **M** | **Y** | | **Y** |  | **D** | **D** | | | **M** | **M** | **Y** | **Y** |
| 1. Did you contact your research nurse?      1. Did you take any antibiotics? |      | | | | |      | | | | | |      | | | | |
| *If yes which antibiotic (i.e. Amoxicillin 250 mg)?* |  | | | | | | | | | | | | | | | |
| *If yes, for how long have you been taking it? (DD.MM.YY – DD.MM.YY)* |  |  |  |  |  | |  | **_** |  |  | | |  |  |  |  |
|  | **D** | **D** | **M** | **M** | **Y** | | **Y** |  | **D** | **D** | | | **M** | **M** | **Y** | **Y** |
|  | Ongoing  | | | | | | | | | | | | | | | |
| 4. Did you take a second lot of antibiotics during this UTI episode?  *(if yes, which antibiotic?)* |    | | | | | | | | | | | | | | | |
|  |  | | | | | | | | | | | | | | | |
| *If yes, for how long have you been taken it? (DD.MM.YY- DD.MM.YY)* |  |  |  |  |  | |  |  |  |  | | |  |  |  |  |
|  | **D** | **D** | **M** | **M** | **Y** | | **Y** |  | **D** | **D** | | | **M** | **M** | **Y** | **Y** |
|  | Ongoing  | | | | | | | | | | | | | | | |
| 5. Did you take more than two antibiotic treatments for this UTI episode?  (if yes, please enter their details below) |  | | | | |  | | | | | |  | | | | |
| *Antibiotic Name* | *DD/MM/YY* | | | | | *DD/MM/YY* | | | | | |  | | | | |
|  |  | | | | |  | | | | | | Ongoing  | | | | |
|  |  | | | | |  | | | | | | Ongoing  | | | | |
|  |  | | | | |  | | | | | | Ongoing  | | | | |
|  |  | | | | |  | | | | | | Ongoing  | | | | |
|  |  | | | | |  | | | | | | Ongoing  | | | | |
| 6. Did you send a urine specimen to the trial laboratory? |  | | | | |  | | | | | |  | | | | |
| 7. Did you return a UTI questionnaire? |  | | | | |  | | | | | |  | | | | |
| 8. Have you seen any blood in your urine? |  | | | | |  | | | | | |  | | | | |
| **Signs and Symptoms- Episode 1** | | | | | | | | | | | | | | | | |
| **(Please tick ‘**✓**’ the appropriate answer)** | **YES** | | | | | **NO** | | | | | | **Don’t know** | | | | |
| a. Urine is cloudy |  | | | | |  | | | | | |  | | | | |
| b. Urine has changed colour | ¨ | | | | |  | | | | | |  | | | | |
| c. Urine smells |  | | | | |  | | | | | |  | | | | |
| d. Blood in urine |  | | | | |  | | | | | |  | | | | |
| e. Bladder discomfort |  | | | | |  | | | | | |  | | | | |
| f. Tummy pain |  | | | | |  | | | | | |  | | | | |
| g. Kidney/back pain |  | | | | |  | | | | | |  | | | | |
| h. Pain in penis (men only) |  | | | | |  | | | | | |  | | | | |
| i. Pain/stinging/burning on passing urine |  | | | | |  | | | | | |  | | | | |
| j. Have to pass urine more than usual (with or without a catheter) |  | | | | |  | | | | | |  | | | | |
| k. Can’t pass urine |  | | | | |  | | | | | |  | | | | |
| l. Feel run down |  | | | | |  | | | | | |  | | | | |
| m. Feel hot |  | | | | |  | | | | | |  | | | | |
| n. Feel anxious |  | | | | |  | | | | | |  | | | | |
| o. Leak urine a bit or more than usual |  | | | | |  | | | | | |  | | | | |
| p. Don’t feel well |  | | | | |  | | | | | |  | | | | |
| q. Autonomic dysreflexia |  | | | | |  | | | | | |  | | | | |
| r. Increased spasticity |  | | | | |  | | | | | |  | | | | |
| s. Vertigo |  | | | | |  | | | | | |  | | | | |
| t. Other *(please state below)* ______________________ |  | | | | |  | | | | | |  | | | | |
| **Actions taken- Episode 1** |  | | | | |  | | | | | |  | | | | |
| What actions have you taken in response to the new/worsening symptoms you described in the previous table?    tick ‘✓’ all the actions taken for UTI episode | **Yes** | | | | | **No** | | | | | | **Don’t know** | | | | |
| a. Taken a urine specimen to the GP |  | | | | |  | | | | | |  | | | | |
| b. Sent a urine specimen to the trial lab in the pot provided |  | | | | |  | | | | | |  | | | | |
| c. Contacted my GP |  | | | | |  | | | | | |  | | | | |
| 1. Contacted my hospital consultant      1. Contacted my research nurse |      | | | | |      | | | | | |      | | | | |
| f. Contacted another healthcare professional  e.g. continence advisor |  | | | | |  | | | | | |  | | | | |
| g. Taken products which I have purchased from my chemist |  | | | | |  | | | | | |  | | | | |
| h. Other including increase fluid intake (please state below) |  | | | | |  | | | | | |  | | | | |
|  |  | | | | |  | | | | | |  | | | | |

| (Please tick ‘✓’ the appropriate answer) | **Yes** | **No** | **Don’t know** |
| --- | --- | --- | --- |

## Section C catheter use

Ask participant to refer to their 7-day catheter-use diary and complete the table below:

|  | Date  (DD/MM/YY) | How many times did you catheterise using |
| --- | --- | --- |
|  |  | a single-use catheter? |
| Day 1 |  |  |
| Day 2 |  |  |
| Day 3 |  |  |
| Day 4 |  |  |
| Day 5 |  |  |
| Day 6 |  |  |
| Day 7 |  |  |

During the last month have you needed a further supply of catheters?

YES 

NO 

If ‘Yes’, did you have to buy catheters or any other items yourself?

YES 

NO 

Not applicable 

| **Item** | **Type and make of product** | **Quantity e.g. box of 100 or number of items** | **Cost (if bought)** |
| --- | --- | --- | --- |
| Catheters |  |  |  |
| Lubricant |  |  |  |
| Anything else related to your catheter |  |  |  |

# Monthly Questionnaire – Intervention group

**Section A Urinary tract infection (SAME AS FOR CONTROL GROUP)**

## Section C catheter use

Ask participant to refer to their 7-day catheter-use diary and complete the table below:

|  | Date  (DD/MM/YY) | How many times did you catheterise using a single-use catheter? | How many times did you catheterise using a reusable catheter? |
| --- | --- | --- | --- |
| Day 1 |  |  |  |
| Day 2 |  |  |  |
| Day 3 |  |  |  |
|  |  |  |  |
| Day 4 |  |  |  |
| Day 5 |  |  |  |
| Day 6 |  |  |  |
| Day 7 |  |  |  |

How many times are you usually re-using each multi-use catheter before throwing it away? Enter number ________

Did you open any new Cliny catheters this week? YES  NO 

If YES, how many? Enter number _______

During the last month have you needed a further supply of **single use catheters?**

YES 

NO 

If ‘Yes’, did you have to buy catheters or any other items yourself?

YES 

NO 

Not applicable 

| **Item** | **Type and make of product** | **Quantity e.g. box of 100 or number of items** | **Cost (if bought)** |
| --- | --- | --- | --- |
| Catheters |  |  |  |
| Lubricant |  |  |  |
| Anything else related  to your catheter |  |  |  |

During the last month have you needed a further supply of **multi-use catheters**?

YES 

NO 

If ‘Yes’, did you have to buy catheters or any other items yourself?

YES 

NO 

Not applicable 

Did you have to get catheters or any other items from your research nurse?

YES 

NO 

Not applicable 

| **Item** | **Type and make of product** | **Quantity e.g. box of 100 or number of items** | **Cost (if bought)** |
| --- | --- | --- | --- |
| Catheters |  |  |  |
| Lubricant |  |  |  |
| Anything else related  to your catheter |  |  |  |


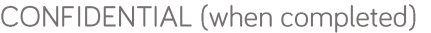


# Health Service Use Schedule


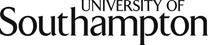


| A non-inferiority randomised controlled trial to compare mixed  (multi/single-use) catheter management with single-use catheter management by intermittent catheter users over 12 months (MultICath)  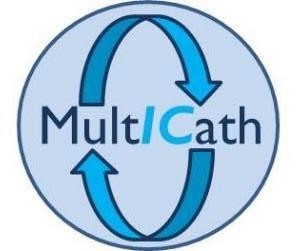  **Health Service Utilisation Questionnaire** | | | | | | | | | | | | | | | | | | |
| --- | --- | --- | --- | --- | --- | --- | --- | --- | --- | --- | --- | --- | --- | --- | --- | --- | --- | --- |
| Unique Study Number | Site No. | | | | | | Study ID | | | | | | | |  |  |  |  |
|  |  | | |  | | |  | |  | |  | |  | |  |  |  |  |
| **Date of Completion** | | |  |  | |  |  | |  | |  | |  | |  | |  |  |
|  |  |  | **D** | **D** | | **M** | **M** | | **Y** | | **Y** | | **Y** | | **Y** | |  |  |

**Month Time of completion**

| Baseline |  |
| --- | --- |
| 6 |  |
| 12 |  |

*Please place a [*✓*} or insert [****requested information****] in appropriate box*

*If you make any errors whilst completing this form, please strikethrough the incorrect data with a horizontal line and initial and date any changes*

**Health Service Utilisation Questionnaire - MultICath**

*Please complete this questionnaire with details of your treatment over the last 6 months.*

*These questions ask about visits to hospital and your GP. Please tick the appropriate boxes and answer the questions where required.*

1. In the last 6 months, have you been admitted to hospital as an inpatient (stayed in hospital overnight or longer)?

Yes  **If Yes, go to Q1a**

No  **If No, go to Q2**

|  |
| --- |

1a. If Yes, how many times did you stay in hospital for one day (or overnight) in the last 6 months?

Enter number of times you stayed in hospital for one day (or overnight)

|  |
| --- |

|  |
| --- |

|  |
| --- |

How many of these one day (or overnight) admissions were for surgery?

| 1b. | If Yes, how many times did you stay in hospital more than one day (or a single night) in the last 6 months?  Enter number of times you stayed in hospital more than one day  How many of these admissions that last more than one day/ night were for surgery? |
| --- | --- |
| 1c. | If Yes, approximately how many nights in total did you spend in hospital in the last 6 months? |

|  |
| --- |

Enter number of nights that you stayed in hospital

1. In the last 6 months, have you had any hospital outpatient appointments (did not stay overnight)?

Yes  **If Yes, go to Q2a**

No  **If No, go to Q3**

2a. If Yes, approximately how many outpatient appointments in total did you have in the last 6 months?

|  |
| --- |

Enter number of times you attended hospital as an outpatient

1. In the last 6 months, have you had to attend the A&E/casualty department but were not admitted overnight?

Yes  **If Yes, go to Q3a**

No  **If No, go to Q4**

3a. If Yes, approximately how many times in total did you attend the A&E/casualty department in the last 6 months?

|  |
| --- |

*Enter number of times you attended the A&E/casualty Department*

1. In the last 6 months, have you had any consultations with a **GP at their practice**?

Yes  **If Yes, go to Q4a**

No  **If No, go to Q5**

4a. If Yes, approximately how many consultations in total did you have with a GP at their practice in the last 6 months?

|  |
| --- |

Enter number of consultations you had with a GP at their practice

1. In the last 6 months, have you had any consultations with a **GP at your home**?

Yes  **If Yes, go to Q5a**

No  **If No, go to Q6**

5a. If Yes, approximately how many consultations in total did you have with a GP at your home in the last 6 months?

|  |
| --- |

Enter number of consultations you had with a GP at your home

1. In the last 6 months, have you had any consultations with a **practice nurse at their practice**?

Yes  **If Yes, go to Q6a**

No  **If No, go to Q7**

6a. If Yes, approximately how many consultations in total did you have with a practice nurse at their practice in the last 6 months?

|  |
| --- |

Enter number of consultations you had with a practice nurse at their practice

1. In the last 6 months, have you had any consultations with a **nurse at your home**?

(E.G. district nurse, specialist nurse, etc.)

Yes  **If Yes, go to Q7a**

No  **If No, go to Q8**

7a. If Yes, approximately how many consultations in total did you have with a nurse at your home in the last 6 months?

|  |
| --- |

Enter number of consultations you had with a nurse at your home

1. In the last 6 months have you had any **telephone consultations** with a health care professional?

Yes  **If Yes, go to 8a**

No  **If No, go to Q9**

8a. If Yes, please indicate what health care professional provided this telephone consultation and approximately how many telephone consultations in total you have had in the past 6 months.

**Health Care Professional** ✓ **if applicable Number of consultations**

|  |
| --- |

|  |
| --- |

GP 

Hospital Doctor 

|  |
| --- |

Nurse 

|  |
| --- |

Other health professional 

If Other please provide details

9. In the last 6 months have you had any **out-of-hours consultations** with a health care professional?

Yes  **If Yes, go to Q10a**

No  **If No, go to Q11**

10a. If Yes, please indicate what health care professional provided this out-of-hours consultation and approximately how many out-of-hours-consultations in total you have had in the past 6 months.

**Health Care Professional** ✓ **if applicable Number of consultations**

|  |
| --- |

GP 

|  |
| --- |

Hospital doctor 

|  |
| --- |

Nurse 

|  |
| --- |

Other health professional 

If ‘Other’ please provide details

11. In the past 6 months have you paid for any private health care and/or personal care?

Yes  **If yes, go to Q11a**

No  **If No, please continue to the end of the questionnaire**

11a. If Yes, please indicate what type of health care you have paid for in the past 6 months and what was the cost of this health care to you.

**What heath care have you paid for?**  **What was the cost of this health care?**

1. £
2. £
3. £

**If you wish to provide any further information, please do so below.**

**Thank you for taking the time to complete this questionnaire**

HEAP Appendix 2 – Example layout for result tables

**Table 1a: Example Unit Costs Table (UK£ sterling, Price Year)**

| **Cost Item** | **Unit Cost (£)** | **Source** |
| --- | --- | --- |
| **Intervention** |  |  |
| Milton© |  |  |
| Soap (PZ Cussons UK) |  |  |
| Cliny intermittent catheterisation kits  (catheter + case + carrying pouch) |  |  |
| Additional Cliny catheters |  |  |
| Optilube lubricating jelly (multi-use) |  |  |
| Single-use catheters |  |  |
| **Primary Care** |  |  |
| GP visits |  |  |
| Practice Nurse |  |  |
| Pharmacist |  |  |
| Hospital Doctor |  |  |
| Hospital Nurse |  |  |
| Therapist (assume psychologist) |  |  |
| **Other** |  |  |
| Medication |  |  |

**Table 1b: Source of resource use and outcome data**

|  | **Source of data from trial documentation** |
| --- | --- |
| **Intervention (number)** |  |
| Milton© | Monthly questionnaire |
| Soap (PZ Cussons UK) | Monthly questionnaire |
| Cliny intermittent catheterisation kits (catheter + case + carrying pouch) | Monthly questionnaire |
| Additional Cliny catheters | Monthly questionnaire |
| Optilube lubricating jelly | Monthly questionnaire |
| single-use catheters | Monthly questionnaire |
| **UTI related-resource use** | Monthly questionnaire |
| GP visit | Monthly questionnaire |
| Nurse visit | Monthly questionnaire |
| Hospital consultant contact | Monthly questionnaire |
| Other HCP visit | Monthly questionnaire |
| Antibiotics for UTI | Monthly questionnaire |
| Urine test | Monthly questionnaire |
| Participant out of pocket expenses related to UTI | Monthly questionnaire |
| **Primary care and community (number)** |  |
| GP visits at practice | Health Service Utilisation Questionnaire (6+12 months) |
| GP visits at home | Health Service Utilisation Questionnaire (6+12 months) |
| GP telephone appointments | Health Service Utilisation Questionnaire (6+12 months) |
| GP out-of-hours appointments | Health Service Utilisation Questionnaire (6+12 months) |
| Practice nurse visits at practice | Health Service Utilisation Questionnaire (6+12 months) |
| Practice nurse visit at home | Health Service Utilisation Questionnaire (6+12 months) |
| Practice nurse telephone appointments | Health Service Utilisation Questionnaire (6+12 months) |
| Practice nurse out-of-hours appointments | Health Service Utilisation Questionnaire (6+12 months) |
| Other HCP telephone appointments | Health Service Utilisation Questionnaire (6+12 months) |
| Other HCP out-of-hours appointments | Health Service Utilisation Questionnaire (6+12 months) |
| **Secondary care (number)** |  |
| Hospital Doctor telephone appointment | Health Service Utilisation Questionnaire (6+12 months) |
| Hospital Doctor out of hours | Health Service Utilisation Questionnaire (6+12 months) |
| A&E | Health Service Utilisation Questionnaire (6+12 months) |
| Outpatient appointments | Health Service Utilisation Questionnaire (6+12 months) |
| Admissions | Health Service Utilisation Questionnaire (6+12 months) |
| **Participant personal costs** |  |
| Participant resource use e.g.  private appointments | Health Service Utilisation Questionnaire (6+12 months) |
| Over the counter purchases | Monthly Questionnaire |

**Table 2: Mean (Standard Deviation) Resource Use and Mean Difference (95% Confidence Interval) in Resource Use per Patient over 12 months for the mixed-use strategy compared to single-use catheters only strategy**

|  | **Mixed-use strategy (n=)** | | **Single-use catheters only strategy (n=)** | | **Mean**  **difference** |
| --- | --- | --- | --- | --- | --- |
|  | **Mean** | **Std dev** | **Mean** | **Std dev** | **(95% CI)** |
| **Intervention (number)** |  |  |  |  |  |
| Milton© |  |  |  |  |  |
| Soap (PZ Cussons UK) |  |  |  |  |  |
| Cliny intermittent catheterisation kits (catheter + case + carrying pouch) |  |  |  |  |  |
| Additional Cliny catheters |  |  |  |  |  |
| Optilube lubricating jelly |  |  |  |  |  |
| single-use catheters |  |  |  |  |  |
| **UTI related-resource use (number)** |  |  |  |  |  |
| GP visit |  |  |  |  |  |
| Nurse visit |  |  |  |  |  |
| Hospital consultant contact |  |  |  |  |  |
| Other HCP visit |  |  |  |  |  |
| Antibiotics for UTI |  |  |  |  |  |
| Urine test |  |  |  |  |  |
| Participant out of pocket expenses related to UTI |  |  |  |  |  |
| **Primary care and community (number)** |  |  |  |  |  |
| GP visits at practice |  |  |  |  |  |
| GP visits at home |  |  |  |  |  |
| GP telephone appointments |  |  |  |  |  |
| GP out-of-hours appointments |  |  |  |  |  |
| Practice nurse visits at practice |  |  |  |  |  |
| Practice nurse visit at  home |  |  |  |  |  |
| Practice nurse telephone appointments |  |  |  |  |  |
| Practice nurse out-ofhours appointments |  |  |  |  |  |
| Other HCP telephone appointments |  |  |  |  |  |
| Other HCP out-of-hours appointments |  |  |  |  |  |
| **Secondary care (number)** |  |  |  |  |  |
| Hospital Doctor telephone appointment |  |  |  |  |  |
| Hospital Doctor out of hours |  |  |  |  |  |
| A&E |  |  |  |  |  |
| Outpatient appointments |  |  |  |  |  |
| Admissions |  |  |  |  |  |
| **Participant personal costs** |  |  |  |  |  |
| Participant resource use  e.g. private appointments |  |  |  |  |  |

This table is for illustrative purposes only.

**Table 3: Mean (Standard Deviation) Cost and Cost Difference (95% Confidence Interval) Per Patient over 12 months for the mixed-use strategy compared to single-use catheters only strategy (in 2022/23 UK pounds sterling)**

|  | **Mixed-use strategy (n=)** | | **Single-use catheters only strategy (n=)** | | **Mean**  **difference** |
| --- | --- | --- | --- | --- | --- |
|  | **Mean** | **Std dev** | **Mean** | **Std dev** | **(95% CI) £’s** |
| **Intervention** | | |  | |  |
| Milton© |  |  |  |  |  |
| Soap (PZ Cussons UK) |  |  |  |  |  |
| Cliny intermittent catheterisation kits  (catheter + case + carrying pouch) |  |  |  |  |  |
| Additional Cliny catheters |  |  |  |  |  |
| Optilube lubricating jelly |  |  |  |  |  |
| single-use catheters |  |  |  |  |  |
| **UTI related-resource use** |  |  |  |  |  |
| GP visit |  |  |  |  |  |
| Nurse visit |  |  |  |  |  |
| Hospital consultant contact |  |  |  |  |  |
| Other HCP visit |  |  |  |  |  |
| Antibiotics for UTI |  |  |  |  |  |
| Urine test |  |  |  |  |  |
| Participant out of pocket expenses related to UTI |  |  |  |  |  |
| **Primary care and community** | | |  | |  |
| GP visits at practice |  |  |  |  |  |
| GP visits at home |  |  |  |  |  |
| GP telephone appointments |  |  |  |  |  |
| GP out-of-hours appointments |  |  |  |  |  |
| Practice nurse visits at practice |  |  |  |  |  |
| Practice nurse visit at home |  |  |  |  |  |
| Practice nurse telephone appointments |  |  |  |  |  |
| Practice nurse out-of-hours appointments |  |  |  |  |  |
| Other HCP telephone appointments |  |  |  |  |  |
| Other HCP out-of-hours appointments |  |  |  |  |  |
| **Secondary care** | | |  | | |
| Hospital Doctor telephone appointment |  |  |  |  |  |
| Hospital Doctor out of hours |  |  |  |  |  |
| A&E |  |  |  |  |  |
| Outpatient appointments |  |  |  |  |  |
| Admissions |  |  |  |  |  |
| **Participant personal costs** |  |  |  |  |  |
| Participant resource use  e.g. private appointments |  |  |  |  |  |
| **Total health care costs** |  |  |  |  |  |

This table is for illustrative purposes only.

**Table 4: Utility and QALYs for** **over 12 months for the mixed-use strategy compared to single-use catheters only strategy**

|  | **Mixed-use strategy (n=)** | | **Single-use catheters only strategy (n=)** | | **Mean difference** |
| --- | --- | --- | --- | --- | --- |
|  | **Mean** | **Std dev** | **Mean** | **Std dev** | **(95% CI)** |
| **EQ-5D-5L Index** |  |  |  |  |  |
| EQ-5D-5L Baseline |  |  |  |  |  |
| EQ-5D-5L 3 months |  |  |  |  |  |
| EQ-5D-5L 6 months |  |  |  |  |  |
| EQ-5D-5L 9 months |  |  |  |  |  |
| EQ-5D-5L 12 months |  |  |  |  |  |
| **QALYs at 12 months** |  |  |  |  |  |
| **EQ-5D VAS** |  |  |  |  |  |
| EQ-5D VAS Baseline |  |  |  |  |  |
| EQ-5D VAS 3 months |  |  |  |  |  |
| EQ-5D VAS 6 months |  |  |  |  |  |
| EQ-5D VAS 9 months |  |  |  |  |  |
| EQ-5D VAS 12 months |  |  |  |  |  |

This table is for illustrative purposes only.

## HEAP Appendix 3 – Consolidated Health Economic Evaluation Reporting Standards 2022 (CHEERS 2022) Statement

|  | **Item** | **Guidance for Reporting** | **Reported in section** |
| --- | --- | --- | --- |
| **TITLE** | | | |
| Title | 1 | Identify the study as an economic evaluation and specify the interventions being compared. |  |
| **ABSTRACT** | | | |
| Abstract | 2 | Provide a structured summary that highlights context, key methods, results and alternative analyses. |  |
| **METHODS** | | | |
| Background and objectives | 3 | Give the context for the study, the study question and its practical relevance for decision making in policy or practice. |  |
| Health economic analysis plan | 4 | Indicate whether a health economic analysis plan was developed and where available. |  |
| Study population | 5 | Describe characteristics of the study population (such as age range, demographics, socioeconomic, or clinical characteristics). |  |
| Setting and location | 6 | Provide relevant contextual information that may influence findings. |  |
| Comparators | 7 | Describe the interventions or strategies being compared and why chosen. |  |
| Perspective | 8 | State the perspective(s) adopted by the study and why chosen. |  |
| Time horizon | 9 | State the time horizon for the study and why appropriate. |  |
| Discount rate | 10 | Report the discount rate(s) and reason chosen. |  |
| Selection of outcomes | 11 | Describe what outcomes were used as the measure(s) of benefit(s) and harm(s). |  |
| Measurement of outcomes | 12 | Describe how outcomes used to capture benefit(s) and harm(s) were measured. |  |
| Valuation of outcomes | 13 | Describe the population and methods used to measure and value outcomes. |  |
| Measurement and valuation of resources and costs | 14 | Describe how costs were valued. |  |
| Currency, price date, and conversion | 15 | Report the dates of the estimated resource quantities and unit costs, plus the currency and year of conversion. |  |
| Rationale and description of model | 16 | If modelling is used, describe in detail and why used. Report if the model is publicly available and where it can be accessed. |  |
| Analytics and assumptions | 17 | Describe any methods for analysing or statistically transforming data, any extrapolation methods, and approaches for validating any model used. |  |
| Characterizing heterogeneity | 18 | Describe any methods used for estimating how the results of the study vary for sub-groups. |  |
| Characterizing distributional effects | 19 | Describe how impacts are distributed across different individuals or adjustments made to reflect priority populations. |  |
| Characterizing uncertainty | 20 | Describe methods to characterize any sources of uncertainty in the analysis. |  |
| Approach to engagement with patients and others affected by the study | 21 | Describe any approaches to engage patients or service recipients, the general public, communities, or stakeholders (e.g., clinicians or payers) in the design of the study. |  |
| **RESULTS** | | | |
| Study parameters | 22 | Report all analytic inputs (e.g., values, ranges, references) including uncertainty or distributional assumptions. |  |
| Summary of main results | 23 | Report the mean values for the main categories of costs and outcomes of interest and summarise them in the most appropriate overall measure. |  |
| Effect of uncertainty | 24 | Describe how uncertainty about analytic judgments, inputs, or projections affect findings. Report the effect of choice of discount rate and time horizon, if applicable. |  |
| Effect of engagement with patients and others affected by the study | 25 | Report on any difference patient/service recipient, general public, community, or stakeholder involvement made to the approach or findings of the study |  |
| **DISCUSSION** | | | |
| Study findings, limitations, generalizability, and current knowledge | 26 | Report key findings, limitations, ethical or equity considerations not captured, and how these could impact patients, policy, or practice. |  |
| **OTHER RELEVANT INFORMATION** | | | |
| Source of funding | 27 | Describe how the study was funded and any role of the funder in the identification, design, conduct, and reporting of the analysis |  |
| Conflicts of interest | 28 | Report authors conflicts of interest according to journal or |  |

Husereau D, Drummond M, Augustovski F, de Bekker-Grob E, Briggs AH, Carswell C, Caulley L,

Chaiyakunapruk N, Greenberg D, Loder E, Mauskopf J, Mullins CD, Petrou S, Pwu RF, Staniszewska S;

CHEERS 2022 ISPOR Good Research Practices Task Force. Consolidated Health Economic Evaluation Reporting Standards 2022 (CHEERS 2022) Statement: Updated Reporting Guidance for Health Economic Evaluations. BMJ. 2022;376:e067975.

The checklist is Open Access distributed in accordance with the terms of the Creative Commons Attribution (CC BY 4.0) license, which permits others to distribute, remix, adapt and build upon this work, for commercial use, provided the original work is properly cited. See: http://creativecommons.org/licenses/by/4.0/.

**REFERENCES**

Bosmans JE, de Bruijne MC, van Hout HP, Hermens ML, Adèr HJ, van Tulder MW. Practical guidelines for economic evaluations alongside equivalence trials. Value Health. 2008;11(2):251-8.

Devlin NJ, Shah KK, Feng Y, Mulhern B, van Hout B. Valuing health-related quality of life: An EQ-5D5L value set for England. Health Economics. 2018;27(1):7-22.

Glick HA, Doshi JA, Sonnad SS, Polsky D. Economic Evaluation in Clinical Trials. Gray A, Briggs AH, editors. Oxford: Oxford University Press; 2007. p. 272.

Faria R, Gomes M, Epstein D, White IR. A Guide to Handling Missing Data in Cost-Effectiveness

Analysis Conducted Within Randomised Controlled Trials. Pharmacoeconomics. 2014 Nov 26;32(12):1157–70

Herdman M, Gudex C, Lloyd A, Janssen M, Kind P, Parkin D, et al. Development and preliminary testing of the new five-level version of EQ-5D (EQ-5D-5L). Qual Life Res. 2011 Dec;20(10):1727–36.

Hernandez Alava M, Wailoo A, Kearns B, Pudney S. Mapping EQ-5D-5L to 3L [Internet]. NICE Decision Support Unit. 2022 [cited 2024 Nov 12]. Available from: https://www.sheffield.ac.uk/nicedsu/methods-development/mapping-eq-5d-5l-3l

Hernández Alava M, Pudney S, Wailoo A. Estimating the Relationship Between EQ-5D-5L and EQ-5D3L: Results from a UK Population Study. Pharmacoeconomics. 2023 Feb;41(2):199–207.

Husereau D, Drummond M, Augustovski F, de Bekker-Grob E, Briggs AH, Carswell C, et al. Consolidated Health Economic Evaluation Reporting Standards 2022 (CHEERS 2022) statement: updated reporting guidance for health economic evaluations. BMJ. 2022;376:e067975.

Jones K et al. Unit Costs of Health and Social Care 2023 [Internet]. PSSRU. 2021 [Accessed 2024 Nov

12]. Available from: <https://www.pssru.ac.uk/unitcostsreport/>

Manca A, Hawkins N, Sculpher MJ. Estimating mean QALYs in trial-based cost-effectiveness analysis: the importance of controlling for baseline utility. Health Economics. 2005;14(5):487-96.

NHS Business Service Authority. Prescription Cost Analysis - England 2022/23 [Internet]. 2023

[Accessed 2024 Nov 12]. Available from: https://www.nhsbsa.nhs.uk/statisticalcollections/prescription-cost-analysis-england/prescription-cost-analysis-england-2022-23

NHS England. 2022/2023 National Cost Collection Data Publication [Internet]. London; 2023 [Accessed 2024 Nov 12]. Available from: https://www.england.nhs.uk/costing-in-the-nhs/nationalcost-collection/

NICE. British National Formulary. [Accessed on 2024 Nov 12]. Available from: https://bnf.nice.org.uk/

NICE. NICE health technology evaluations: the manual (PMG 36) [Internet]. 2022. Available from: https://www.nice.org.uk/process/pmg36/chapter/introduction-to-health-technology-evaluation

Prieto JA, Murphy CL, Stewart F, Fader M. Intermittent catheter techniques, strategies and designs for managing long-term bladder conditions. Cochrane Database Syst Rev. 2021;10(10):CD006008. Published 2021 Oct 26. doi:10.1002/14651858.CD006008.pub5

von Hippel PT. Regression with Missing Ys: An Improved Strategy for Analyzing Multiply Imputed Data. Sociol Methodol. 2007 Aug 23;37(1):83–117.

Whitehurst DG, Bryan S. Trial-based clinical and economic analyses: the unhelpful quest for conformity. Trials. 2013;14:421. Published 2013 Dec 5. doi:10.1186/1745-6215-14-421.

Willan AR, Briggs AH, Hoch JS. Regression methods for covariate adjustment and subgroup analysis for non-censored cost-effectiveness data. Health Econ. 2004 May 1;13(5):461–75.

**Supplementary table 7**: Completed consolidated Health Economic Evaluation Reporting Standards (CHEERS)

|  | **Item** | **Guidance for Reporting** | **Reported in section** |
| --- | --- | --- | --- |
| **TITLE** | | | |
| Title | 1 | Identify the study as an economic evaluation and specify the interventions being compared. | Title |
| **ABSTRACT** | | | |
| Abstract | 2 | Provide a structured summary that highlights context, key methods, results and alternative analyses. | Abstract |
| **METHODS** | | | |
| Background and objectives | 3 | Give the context for the study, the study question and its practical relevance for decision making in policy or practice. | Intro |
| Health economic analysis plan | 4 | Indicate whether a health economic analysis plan was developed and where available. | Methods |
| Study population | 5 | Describe characteristics of the study population (such as age range, demographics, socioeconomic, or clinical characteristics). | Methods |
| Setting and location | 6 | Provide relevant contextual information that may influence findings. | Methods |
| Comparators | 7 | Describe the interventions or strategies being compared and why chosen. | Intro and methods |
| Perspective | 8 | State the perspective(s) adopted by the study and why chosen. | Methods |
| Time horizon | 9 | State the time horizon for the study and why appropriate. | Methods |
| Discount rate | 10 | Report the discount rate(s) and reason chosen. | Methods |
| Selection of outcomes | 11 | Describe what outcomes were used as the measure(s) of benefit(s) and harm(s). | Methods |
| Measurement of outcomes | 12 | Describe how outcomes used to capture benefit(s) and harm(s) were measured. | Methods |
| Valuation of outcomes | 13 | Describe the population and methods used to measure and value outcomes. | Methods |
| Measurement and valuation of resources and costs | 14 | Describe how costs were valued. | Methods |
| Currency, price date, and conversion | 15 | Report the dates of the estimated resource quantities and unit costs, plus the currency and year of conversion. | Methods |
| Rationale and description of model | 16 | If modelling is used, describe in detail and why used. Report if the model is publicly available and where it can be accessed. | N/A |
| Analytics and assumptions | 17 | Describe any methods for analysing or statistically transforming data, any extrapolation methods, and approaches for validating any model used. | Methods |
| Characterizing heterogeneity | 18 | Describe any methods used for estimating how the results of the study vary for sub-groups. | Methods |
| Characterizing distributional effects | 19 | Describe how impacts are distributed across different individuals or adjustments made to reflect priority populations. | N/A |
| Characterizing uncertainty | 20 | Describe methods to characterize any sources of uncertainty in the analysis. | Methods |
| Approach to engagement with patients and others affected by the study | 21 | Describe any approaches to engage patients or service recipients, the general public, communities, or stakeholders (e.g., clinicians or payers) in the design of the study. | Methods |
| **RESULTS** | | | |
| Study parameters | 22 | Report all analytic inputs (e.g., values, ranges, references) including uncertainty or distributional assumptions. | Methods |
| Summary of main results | 23 | Report the mean values for the main categories of costs and outcomes of interest and summarise them in the most appropriate overall measure. | Results |
| Effect of uncertainty | 24 | Describe how uncertainty about analytic judgments, inputs, or projections affect findings. Report the effect of choice of discount rate and time horizon, if applicable. | Results |
| Effect of engagement with patients and others affected by the study | 25 | Report on any difference patient/service recipient, general public, community, or stakeholder involvement made to the approach or findings of the study | Not reported |
| **DISCUSSION** | | | |
| Study findings, limitations, generalizability, and current knowledge | 26 | Report key findings, limitations, ethical or equity considerations not captured, and how these could impact patients, policy, or practice. | Discussion |
| **OTHER RELEVANT INFORMATION** | | | |
| Source of funding | 27 | Describe how the study was funded and any role of the funder in the identification, design, conduct, and reporting of the analysis | Acknowledgements |
| Conflicts of interest | 28 | Report authors conflicts of interest according to journal or | Declarations of interest |

Source: Husereau D, Drummond M, Augustovski F, de Bekker-Grob E, Briggs AH, Carswell C, et al. Consolidated Health Economic Evaluation Reporting Standards 2022 (CHEERS 2022) statement: updated reporting guidance for health economic evaluations. BMJ. 2022;376:e067975.
